# Child labor and associated risk factors in the wake of the COVID-19 pandemic: a scoping review

**DOI:** 10.3389/fpubh.2023.1240988

**Published:** 2024-02-01

**Authors:** Rima R. Habib, Moussa El Khayat, Joly Ghanawi, Reem S. Katrib, Layal Hneiny, Dana A. Halwani

**Affiliations:** ^1^Faculty of Health Sciences, Department of Environmental Health, American University of Beirut, Beirut, Lebanon; ^2^Wegner Health Sciences Library, University of South Dakota, Sioux Falls, SD, United States

**Keywords:** child labor, COVID-19, pandemic, risk factors, scoping review

## Abstract

Child labor can significantly impact the health, welfare, and development of children engaged in labor. The spread of child labor around the globe is predicted to accelerate as a consequence of the COVID-19 pandemic. To this end, a scoping review was conducted to (a) synthesize emerging themes and results from recent research on child labor during the COVID-19 pandemic, (b) identify factors that increase the risk of children falling into child labor and (c) provide recommendations that can inform the development of policies and programs to ensure that previous efforts to combat child labor are not lost. Six electronic databases (Medline, EMBASE, Scopus, CINAHL, Global health, and Web of Science) were searched on January 21, 2022. The database searches, along with the grey literature search, identified 5,244 studies, of which 45 articles were included in the final review. Several of those articles (8 of 45 articles) reviewed concluded that the pandemic could increase child labor worldwide including the worst forms of child labor. The reviewed studies identified primary risk factors for child labor during the COVID-19 pandemic including economic challenges, temporary school closure and a greater demand for child labor, mortality among parents, and limited social protection. This scoping review identified the need for more field research on child labor following the COVID-19 pandemic to detect emerging patterns of child labor and to develop effective intervention measures. There is also a need for further empirical research on the consequences of the COVID-19 pandemic on gender differences in occupational exposure and health outcomes among working children and marginalized groups such as migrants, refugees, and minority groups. Based on the conclusions drawn from this review, it is evident that addressing child labor in the wake of the pandemic necessitates a multi-sectoral response by the government, businesses, civil society, and funding/donor agencies. This response should address various areas such as education, social and child protection, and legislation to support vulnerable children and their families in order to combat child labor subsequent to the pandemic.

## 1 Introduction

Child labor refers to the act of exploiting children by engaging them in any form of work that deprives them of their childhood, hinders their ability to regularly attend school, and negatively affects their health and development (1). The International Labor Organization (ILO) defines child labor as work that deprives children under the age of 18 of their childhood, potential and dignity, while also posing risks to their physical and/or mental development (2). According to the ILO, child labor has increased to 160 million children in 2020, accounting for 1 in 10 children worldwide (3). Historically, child labor has been a pervasive issue worldwide especially among marginalized populations particularly in low and middle income countries, where it is often deeply ingrained in social and economic dynamics (3). Although progress to combat child labor has derailed since 2016, over the past two decades the number of children engaged in child labor globally has declined significantly (4). This progress has particularly stalled in low-income countries, where child labor is especially prevalent (5). Progress toward the eradication of child labor is integral to achieving the Sustainable Development Goal (SDGs), particularly achieving SDGs relating to decent work and economic growth (6, 7).

Based on the international literature, the major factors contributing to child labor include socio-demographic and economic factors such as poverty, displacement, large family size, cultural beliefs, and parental educational status (8, 9). Families in underprivileged populations depend on their children to provide income as a means for survival (8). Consequently, children undertake hazardous jobs in sectors such as domestic work, agriculture, construction, fishing, mining, and manufacturing, where working conditions are precarious and challenging (3).

Child labor has been strongly associated with different adverse social and health consequences. Negative health outcomes have been reported among working children such as injuries, musculoskeletal disorder symptoms, malnutrition, respiratory disease, as well as premature death (10–13). According to ILO, approximately 79 million children were involved in hazardous work in 2020 (3). ILO also estimates that 10 million children had work-related injuries while 20,000 died as a result of child labor in 2013 (14). Children are particularly vulnerable to occupational hazards since they are still developing mentally and physically (15). Children’s psychological health can be negatively impacted when exposed to abusive relationships with employers or clients (16, 17). In addition, working children are vulnerable to exploitation, trafficking, and forced labor (18). Child labor also affects children’s access to education and a decent standard of living (5).

It was anticipated that the impacts of the COVID-19 pandemic and related public health measures have accelerated the rise of child labor globally. This is especially true as the pandemic has aggravated the risk factors associated with child labor through its effects on the economy and the withdrawal of children from schooling (19). An estimated 8.9 million children were predicted to enter the workforce by the end of 2022 due to the pandemic (3). In addition, the working conditions for children already involved in child labor were predicted to worsen including hazardous working conditions and trafficking and violence (20). The consequences of the increase in child labor caused by the pandemic could last generations due to diminished access to education and school closures during the pandemic lockdown, worse employment opportunities, and the development of chronic health issues among working children (19).

Moreover, children are under increased pressure to enter the workforce due to a rise in poverty caused by the economic effects of the pandemic (21, 22). As of March 2023, the global poverty rate estimated by the World Bank is 46.9%, representing 44 million people living in poverty (23). Moreover, the mortality of parents or caregivers due to COVID-19 forces children to enter the workforce to be able to support themselves and their families (24). Children in developing countries are especially vulnerable to fall into child labor due to lack of social protection systems and the presence of a large informal sector (21).

To our knowledge, this is the first scoping review that aims to summarize the existing knowledge regarding the impacts and repercussions of the COVID-19 pandemic on child labor, as well as guide future research and inform policies to protect vulnerable children. The expected effects of COVID-19 on child labor require urgent attention and should be given the utmost priority. In addition, it is necessary to develop measures and strategies that can mitigate the effects of the pandemic on children, as well as the threat it poses to the progress made in combating child labor. This review aims to (a) synthesize emerging themes and results from recent research on child labor during the COVID-19 pandemic, (b) identify factors that increase the risk of children falling into child labor and (c) provide recommendations which can inform the development of policies and programs to ensure that previous gains against child labor will not be lost.

## 2 Materials and methods

### 2.1 Search strategy

A scoping review was conducted following the PRISMA extension for Scoping Reviews (PRISMA-ScR) to map the available literature on this topic (25). The search strategy was prepared with the assistance of a medical librarian using a combination of key words on two main concepts (1) child labor and (2) the COVID-19 pandemic. A review of 6 selected electronic databases including Medline, Embase, Scopus, CINAHL, Global health, and Web of Science was conducted on January 21, 2022. The search strategies for each database are presented in the Supplementary Table S1.

In addition, we manually searched the websites of international agencies (ILO, UNICEF, UNICEFUSA, WHO, and UNDP) to identify relevant articles.

### 2.2 Selection process

Articles that met the following three criteria were included in this review:

- Study population includes working children, that is working children younger than 18 years old as defined by ILO, or that included workplaces or communities employing children;
- Study context is focused on the COVID-19 pandemic;
- Study design includes both primary and secondary studies including reviews, letters, and editorials. We excluded press releases and conference abstracts. No restrictions were placed on language and publication date (i.e., our review identified articles published in all languages and irrespective of their publication dates; however, as COVID-19 was a keyword in the search strategies, the identified studies were published during or after the COVID-19 pandemic – until January 21, 2022).

Endnote was used to import the search results of all databases. Duplicates were removed automatically by Endnote and manually by the reviewers. Two reviewers worked separately and in duplicates to screen first the title and abstracts and then the full text of the articles obtained from the databases, guided by pre-established eligibility criteria. The screening questions were re-examined and validated through a calibration exercise conducted by the two reviewers.

### 2.3 Data abstraction and analysis

Data abstraction was conducted by one of the reviewers using a data abstraction table form, which was prepared by the authors of this review. Data on the following were extracted from each included article: (1) country, (2) data collection methods, (3) sample size, (4) age/gender, (5) occupation, (6) school enrollment/grade attained, (7) working conditions, (8) prevalence of child labor, (9) distribution of child labor by field of work, age, and gender, (10) reasons for engaging in child labor, (11) risk factors and (12) policy implications.

## 3 Results

### 3.1 Search results

**Figure 1:**
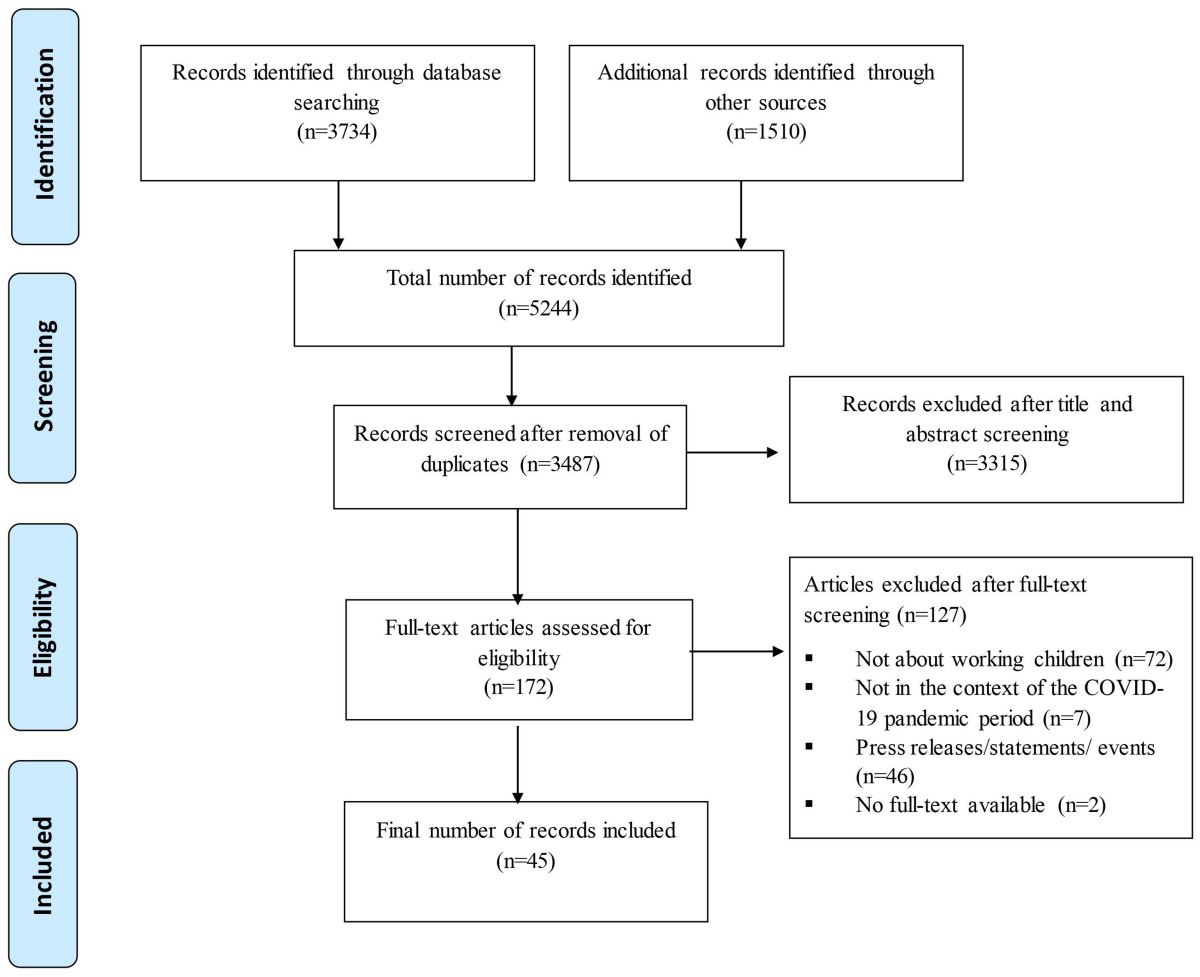
PRISMA flow diagram. The below diagram represents the study selection process from “Identification” of relevant studies (above) to what was “Included” (below).

The PRISMA flow diagram (Figure 1) below shows the studies selection process. We retrieved 3,734 studies from database search while 1,510 studies were retrieved by screening the grey literature. After title and abstract reviewing, 3,315 studies were found to be ineligible while 172 studies were included. After full-text screening of the 172 studies included in the first stage of screening, 45 articles^1^ were included while 127 studies were excluded.

### 3.2 Characteristics of included studies

**Figure 2:**
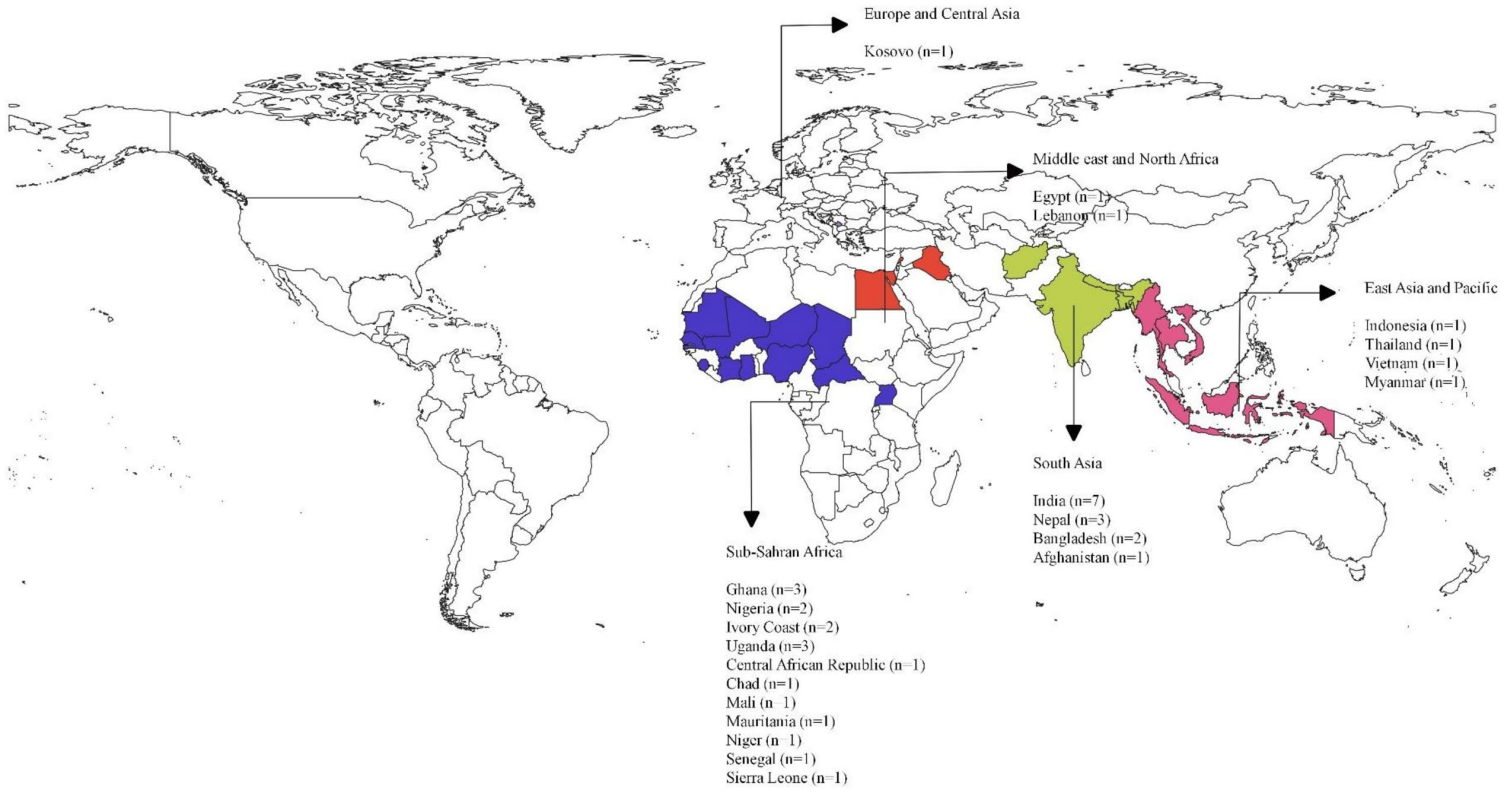
Geographical distribution of included studies (*n* = 29). This figure represents the geographical distribution of the studies included within this scoping review, with purple representing countries in Sub-Saharan Africa, red representing the Middle East and North Africa Region, green representing South Asia, and pink representing East Asia and Pacific.

Of the 45 studies included in this review, 29 studies focused on individual countries including India (*n* = 7;16%), Nepal (*n* = 3;7%), Ghana (*n* = 3;7%), Uganda (*n* = 3;7%), Ivory Coast (*n* = 2;4%), Bangladesh (*n* = 2;4%) and Nigeria (*n* = 2;4%) (Table 1). The remaining studies focused on child labor globally (*n* = 14;31%) or on specific regions - South Asia and Latin America (*n* = 2;4%). Figure 2 shows the geographical distribution of the included studies focusing on specific countries.^2^ The type of articles included were literature reviews (*n* = 22; 49%), letters (*n* = 2;4%), commentary/viewpoint (*n* = 2;4%), feature/news article (*n* = 3;7%), quantitative studies (*n* = 7;16%), and qualitative studies (*n* = 5;11%) (Table 1).

**Table 1:** Descriptive characteristics of included studies the below table categorizes the included studies into three separate descriptive characteristics: “Country,” “study design/data collection methods,” and “theme(s).

Descriptive characteristic	*N*	%
Country^a^		
India	7	16
Nepal	3	7
Ghana	3	7
Uganda	3	7
Ivory Coast	2	4
Bangladesh	2	4
Nigeria	2	4
Egypt	1	2
Indonesia	1	2
Iraq	1	2
Lebanon	1	2
Kosovo	1	2
Afghanistan	1	2
Vietnam	1	2
Myanmar	1	2
Thailand	1	2
Central African Republic	1	2
Chad	1	2
Mali	1	2
Mauritania	1	2
Niger	1	2
Senegal	1	2
Sierra Leone	1	2
Study design/data collection methods		
Literature Review	22	49
Letter	2	4
Commentary/viewpoint	2	4
Feature/news article	2	4
Quantitative Study	8	16
Qualitative Study	5	11
Case study	4	9
Theme(s)		
Risk factors for child labor	39	87
Policy recommendations to combat child labor	33	73
Effect of COVID-19 on prevalence of child labor	8	17
Effect of the COVID-19 on vulnerable populations (refugees, minority groups)	3	7
Assessment of gender differences	3	7

Each category is listed based on the order of the characteristic with the highest number of included studies to the lowest.

The majority of articles (*n* = 39,87%) described risk factors associated with child labor during the pandemic (3, 19–22, 24, 26–55), while 30 (73%) studies provided policy recommendations to combat child labor during the COVID-19 pandemic (3, 19, 20, 22, 24, 27–29, 31–36, 38, 41, 42, 44–50, 55–60). Six studies measured changes in child labor prevalence after the beginning of the COVID-19 pandemic (26, 31, 36, 38, 47, 51). In addition, two studies conducted simulations to estimate the increase in child labor prevalence due to the pandemic (3, 28). Few studies focused on children in marginalized minority groups, migrants, and refugees in the reviewed studies (45, 47, 58). Moreover, three studies assessed gender differences in child labor prevalence or reasons for entering the labor force (26, 31, 47) (Table 1).

### 3.3 Effects on the prevalence of child labor during and after the COVID-19 pandemic

Several of the reviewed studies have concluded that the pandemic threatens an increase in child labor worldwide (see Table 1 for further details). The studies also reported an increase in the worst forms of child labor such as hazardous work and bonded labor which occurs when vulnerable families are exploited by lenders who offer credit with terms that amount to debt bondage (24, 33). Based on one study which simulated child labor trends, it was projected that approximately 9 million children would be forced into child labor by the end of 2022 due to rising poverty resulting from the pandemic (61). Among these children, more than half (4.9 million) are anticipated to be young children between the ages of 5–11 (61). Another study conducted by Economic Commission for Latin America and the Caribbean (ECLAC) and ILO estimated that the pandemic could lead to the entry of between 109,000 and 326,000 children and adolescents into the labor market in Latin America and the Caribbean, adding to the 10.5 million children that are already involved in child labor in the region (62). This is consistent with several reviewed primary studies which showed an increase in child labor after the beginning of the pandemic. One study assessed the impact of COVID-19 on child labor in cocoa-growing households in Côte d’Ivoire. The study found a surge of 21.5% in number of children engaged in hazardous child labor between March 2020 and May 2020 (38). Furthermore, a study in Indonesia found that 63% of students began working in the agricultural sector during the pandemic while only 37% had been working in that sector prior to pandemic (26). A global study conducted by Save the Children among 8,068 child respondents found that 2.3% of boys and girls reported getting paid for work (47). While another study found that children in Egypt were sent to work in agricultural work including cotton cultivation after the start of the pandemic (35).

### 3.4 Risk factors associated with child labor during the COVID-19 pandemic

**Figure 3:**
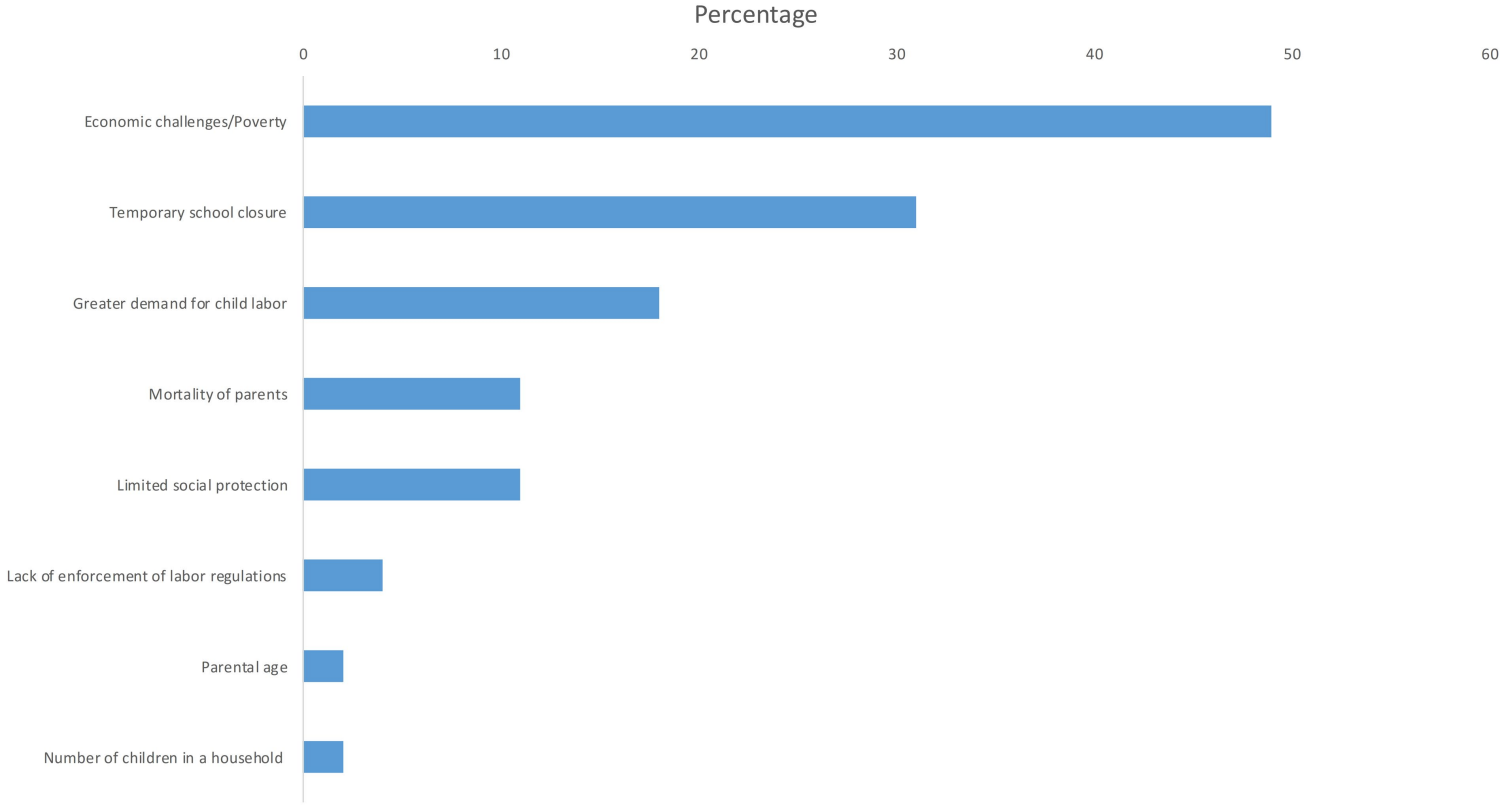
Risk factors for child labor during the COVID-19 pandemic era. The below chart presents the percentage of each risk factor for child labor during the COVID-19 pandemic era throughout the studies included.

Various factors related to the COVID-19 pandemic were identified as contributing risk factors to child labor. The primary risk factors identified in the reviewed studies include economic challenges (49%), temporary school closure (31%), a greater demand for child labor (18%), mortality among parents (11%), and limited social protection (11%) (Figure 3). Other factors include lack of enforcement of labor regulations (4%), parental age (2%) and number of children in a household (2%) (Figure 3).

The reviewed literature suggests that the economic impact of the COVID-19 pandemic is the primary cause for the rise in child labor (3, 19, 20, 24, 26–32, 34, 35, 46–51, 53, 54, 63) (Figure 3). In addition, several primary studies found that poverty pushed children to start working to support their families (8, 27, 51). A global study found that children belonging to households that had lost over half of their income were 1.3 times more inclined to report being involved in paid work (i.e., engaging in child labor) compared to children from households that had not faced similar income loss (47). A three-country study in Uganda, Nepal and Ghana which included 81 children portrayed that restrictions imposed by the pandemic negatively affected livelihoods of poor families and increased the pressure on children to work in hazardous working conditions (27). A study conducted in India found that around half of the children were pushed into child labor due to the economic distress in their families caused by the pandemic (51). In addition, greater food insecurity caused by the pandemic also resulted in an increase in child labor (27, 37, 53).

A number of publications identified that school closure due to the pandemic has caused an increase in child labor (3, 19, 20, 22, 24, 30, 35, 41, 43, 44, 50, 52, 55, 62) (Figure 3). In one study, children reported that their presence at home due to school closure resulted in the expectation that they should work to support their families, which renders distance learning challenging (52). An additional factor identified in the reviewed studies is the loss of parents or caregivers which increases their vulnerability to child labor, trafficking, and other forms of exploitation (21, 24, 34, 41). One study found that some children in India who started working during the pandemic reported that their parents were not allowing them to return to school now that they are contributing to the family income (64).

In addition, the COVID-19 pandemic has caused an increased demand for child labor as employers may be more likely to hire child laborers to decrease costs and cope with economic slowdown (21, 30, 31, 37, 40, 41, 45, 46) (Figure 3). A case study in India found that employers had increased demand for children as a source of cheap labor leading to greater cases of child trafficking (30). In addition, a study in Lebanon also found that farms resorted to child labor as a source of cheap labor as a result of the pandemic (31). Furthermore, the restrictions placed on movement and gatherings led parents to rely on children to assist in agriculture during harvesting and marketing season, as they are less likely to be caught by law enforcement (41).

According to one global study, the age of parents or caregivers and the number of children in a household were linked with the proportion of girls and boys who felt burdened by excessive chores, preventing them from attending school (47). Only 8% of children with parents/caregivers aged 18–24 years old reported having too many chores, while 17% of children with parents/caregivers aged 50–59 years reported too many chores (47). Furthermore, in households with only one child, 6% of children reported having too many chores to attend school, whereas this figure rose to 27% of the children living in households with more than 6 children (47).

Furthermore, the absence of adequate social protection or child protection services were identified as factors which can exacerbate the economic effect of the pandemic on children (20, 21, 39, 40, 48) (Figure 3). In addition, weak enforcement of labor regulations due to movement restriction or diversion of resources was also found to increase the employment of children (19, 50). Another risk factor identified in the reviewed studies is parental death or sickness due to COVID-19; whereby the loss of adult wage earners increases pressure on children to work to support their families (21, 41, 44, 50, 55) (Figure 3).

### 3.5 Prevalence of child labor by gender

One study found that the reason for children entering the workforce differed by gender; males were more likely to enter the workforce to take advantage of available time in their daily lives, whereas females were more likely to enter the workforce to help their parents (26). Another study conducted in Lebanon found that boys are assigned significantly more tasks than girls in in the agricultural sector which increases their vulnerability to various occupational hazards (31). One study examined the effect of COVID-19 pandemic on children’s work at home. 63% of girls reported they are doing more chores within the house compared to 43% of boys (47). Furthermore, 20% of girls reported that they have a significant amount of chores to do to be able to go to school, compared to 10% of boys (47).

The findings of a global study show that more than half (54%) of the children surveyed reported an increase in their chores during the COVID-19 pandemic (47). Another study in Myanmar found that 83% of children reported an increase in domestic work at home (36).

### 3.6 Vulnerable populations

A few of the reviewed studies focused on the case of children in marginalized minority groups, migrants, and refugees (45, 47, 58). A global study conducted by Save the Children found that children from minority groups in East and Southern Africa had nearly twice (4.1%) the likelihood of being engaged in paid work than children who did not belong to a minority group (2.1%) (47). One study focused on children of seasonal migrant workers in Nepal and the potential effects of COVID-19 on their lives (29). The study found that children of migrant workers are more prone to enter child labor or engage with household work, which affects their ability to continue their education (29). One study identified various barriers faced by migrant children including social stigma, discrimination, and lack of access to social services (example: healthcare and education), which has been aggravated by the pandemic (45). Children are also motivated by the need to earn additional income to support their families’ and afford basic necessities (45).

### 3.7 Working conditions and health outcomes

Only 3 studies examined working conditions for child laborers. None of the reviewed studies measured health outcomes among working children during the COVID-19 pandemic. One study conducted in Ghana, Nepal, and Uganda found poor working conditions for working children (27). More than one-third of the children interviewed were engaged in work for a minimum of 10 hours a day, occasionally working seven days a week, while others worked 16 hours a day (27). Children in Ghana provided accounts of their work in goldmines, which included tasks such as carrying heavy loads, breathing dust from processing machines, crushing ore with hammers, and handling mercury. Children also reported exploitation, violence, sexual harassment, and payment theft (27). Another study conducted in India found that children had unhygienic and unclean working environments, extreme working hours, lack of food, and inability to access the toilet (51). Moreover, a study in Lebanon reported that children between the ages of 5–11 years were involved in hazardous tasks such as pesticide application and driving machinery, leading to allergic reactions to chemicals, swelling, sprains and strains, cuts and bruises, and vomiting and dizziness (31).

### 3.8 Policy implications

The included studies provided various recommendations at different levels and in different areas to combat the impact of the pandemic on child labor. In light of this scoping review, the call for actions required to combat child labor included calls for a multi-sectoral response by States, businesses, and civil society, and donors – such as USAID, ILO, and the World Bank – to support vulnerable children and their families.

At the government level, recommendations pertain to providing social protection measures as well as improving access to healthcare, education, living wages, and food security. Several studies recommend implementing cash transfer programs to help families experiencing, or susceptible to, living in poverty (22, 27, 35, 49, 56, 59) (Table 2). One study examined the impacts of an unconditional cash transfer program on hazardous child labor in cocoa-growing households in Ghana (56). The study found that cash transfer has reduced child labor prevalence by 9.3% (56). However, the program had no effect on the severity of child labor measured by the number of hazardous tasks undertaken by the children (56). Two studies recommended the implementation of an in-kind or conditional cash transfer, which would require families to send their children to school where they receive food to take home to their families (49, 59) (Table 2). In addition, families of children at risk of child labor should be provided with food assistance and emergency livelihoods programs (20) (Table 2).

**Table 2:** Recommendations for combating child labor during COVID-19 pandemic in the reviewed studies as per three levels of action.

Level of action	Recommendations	Studies
Governments		
Social protection	Implement a cash transfer program Provide food assistance Provide in-kind or conditional cash transfer	Ahad et al. (22), Becker (27), ILO (35), Schmidt and Uyeda (49), International Cocoa Initiative (56), and Azqueta et al., (59) The Alliance for Child Protection in Humanitarian Action (20) Schmidt and Uyeda (49) and Azqueta et al., (59)
Child protection	Strengthen child protection services	Ritz et al. (47) and ILO (61)
Policies and legislation	Enforce and strengthen child labor standards Strengthen labor regulation in global supply chains	ILO and UNICEF (3), The Alliance for Child Protection in Humanitarian Action (20), and Cepal (62) Becker (27), ILO (33), Ramani and van Uden (45), and ILO (61)
Educational and financial support	Promote universal access to education Provide financial support for families Improve educational systems Implement a comprehensive outreach effort Strengthen school monitoring systems	ILO and UNICEF (3), Ahad et al. (22), ILO and UNICEF (24), Becker (27), ECLAC and ILO (28), Daly et al. (29), Kundu (41) Kundu (41), Pattanaik (44), and Ritz et al. (47) ILO and UNICEF (3), UNICEF (19), Becker (27), Schmidt and Uyeda (49), and UNICEFUSA (50) The Alliance for Child Protection in Humanitarian Action (20) The Alliance for Child Protection in Humanitarian Action (20)
Businesses		
Reduce risks of child labor	Reduce risks of child labor in domestic and global supply chains Provide access to social protection Establish grievance redressal mechanisms	ILO and UNICEF (3) and Becker (27) Becker (27) Becker (27)
Civil society and donors		
Public awareness	Raise public awareness	The Alliance for Child Protection in Humanitarian Action (20), Ahad et al. (22), ILO (36), Mutashar et al. (42), Kechagia and Metaxas (57), and Tran and Journal (60)
Technical and financial support	Provide support for social protection, child protection and public education programs Support mechanisms for promoting accountability for allocated funds Establish capacity building programs	Becker (27) and Ramaswamy and Seshadri (46) Becker (27) Larmar et al. (58)

Seven of the reviewed studies identified that universal access to education is necessary to prevent children from entering the workforce (3, 22, 24, 27–29, 41) (Table 2). Three of the reviewed studies suggested the need for governments to provide assistance to parents or caregivers in facilitating their efforts to send children back to school including through the relaxation of school fees or strengthening community-based child protection mechanisms to identify children who are at risk of discontinuing their schooling and entering child labor (41, 44, 47) (Table 2). Furthermore, various studies indicate that improving the education systems can be beneficial in reducing the number of working children (3, 19, 27, 49, 50) (Table 2). Education programs should also be customized to the specific needs of children of different age groups, girls and boys, children with disabilities, migrant, displaced and refugee children, as well as other vulnerable children at risk of child labor (20, 41). In addition, it is advisable to implement a comprehensive outreach initiative to guarantee enrolment of all children who have not concluded compulsory schooling requirements, as well as to develop school monitoring systems to identify children at-risk of dropping out or who dropped out in order to engage in child labor (20, 47) (Table 2).

Four of the reviewed studies provided recommendations to be implemented by governments and businesses to prevent child labor in the supply chain especially in order to combat the effect of the COVID-19 pandemic on child labor. The reviewed literature identified the need for governments to ensure that national legislation addressing child labor is in line with international standards and to enforce these standards (Table 2) (3, 20, 27, 28). Governments should be required to enforce adequate legislative protections and enforcement to reduce the risks faced by children involved in domestic work (3). Governments should enact legislation mandating companies to conduct comprehensive human rights assessments throughout their global supply chains to ensure they do not contribute to child labor (3, 27, 33, 45) (Table 2). Additionally, two of the reviewed articles identified the need for businesses to implement measures to ensure they are not contributing to child labor throughout their supply chains (3, 27). One study advocated for businesses to provide access to social protection schemes and support to poor and vulnerable families as well as establish workplace-based grievance redressal mechanisms (27) (Table 2).

Various studies identified the need for civil society leaders and organizations to support the efforts of governments and businesses in fighting child labor. A number of reviewed studies recommended the need for civil society to work toward raising public awareness on the effects of child labor (20, 22, 36, 42, 57, 60) (Table 2). Moreover, two studies have stressed the need for donors such as international donor agencies – such as World Bank, USAID, ILO, etc. - to support efforts to combat child labor (27, 58) (Table 2). One study recommended that donors should provide support both technical and financial, for programs targeting the enhancement of social protection, child protection, and public education (27, 46) as well as supporting mechanisms for promoting accountability allocated funds (27) (Table 2). Furthermore, it is essential to introduce capacity building programs that strengthen collaboration among social workers, non-governmental organizations, health professionals, and community-based organizations to safeguard vulnerable children amidst the challenges posed by the pandemic (58) (Table 2).

## 4 Discussion

This is the first scoping review that attempts to summarize the existing knowledge regarding the effects of the COVID-19 pandemic on child labor, guide future research, and inform policies to protect vulnerable children. Various risk factors detrimental to children being pushed into labor were identified including rising poverty, temporary school closure, loss of parents or caregivers to COVID-19, and greater demand for child labor among employers. Furthermore, various measures were identified to fight child labor at different sectoral levels including education, social protection, child protection and policies, and legislation.

### 4.1 Prevalence and risk factors associated with child labor

The reviewed literature predicts that the impact of the COVID-19 pandemic and public health measures will accelerate the prevalence of child labor leading to adverse health and social consequences on children worldwide. Some of the reviewed studies indicated that the COVID-19 pandemic contributes to deteriorating the working conditions for child laborers such as longer working hours (27). Children who experience longer working hours are more likely to exhibit worse physical outcomes, such as acquiring an injury or falling ill (65, 66). In addition, the reviewed literature also reported that the likelihood of abuse increased with longer working hours (65). The literature additionally presents evidence of new cases of bonded child labor, as desperate families offer their children’s labor to acquire loans or to service existing ones from predatory lenders (33). Bonded labor is associated with some of the worst forms of child labor, including domestic servitude, commercial sexual exploitation, and hazardous work (33).

Some of the reviewed studies also show that the COVID-19 pandemic has amplified the vulnerability of migrant and minority children to child labor. One of the reviewed studies reported that children of migrant workers are more likely to enter into child labor or engage with household work which affects their ability to continue their education (29). According to UNICEF, 36.5 million children were displaced from their homes by the end of 2021 as a result of conflict, violence, and other crises (67, 68). Children who migrate alone are at a high risk of falling into child labor or being trafficked for sexual exploitation (69). Child labor rates in countries facing conflict, fragility or disaster are 77% higher than global averages while the incidence of hazardous work is 50% higher (4). Rapid assessments and ongoing monitoring are required to examine the effect of the pandemic on the risk of child labor in disaster and conflict settings (33).

The COVID-19 pandemic has exacerbated the domestic work burden on children especially girls due to greater demand for household chores and caring responsibilities (47). This greater burden of chores is dependent on the parent or caregivers’ ages, as well as the number of children in the household (47). National and global action plans often overlook domestic work and are beyond the scope of labor inspectorates, thereby leaving children, particularly girls highly susceptible to abuse and exploitation (3). Girls between the ages of 5 and 14 years old are likely to spend between 40% more time than boys in household chores compared to boys their age (70). Social norms related to girls’ school attendance and use of time can diminish their access to education and increase their risk of falling into child labor (71). Governments and community-based organizations should actively advocate for gender equality by implementing interventions that promote social and behavioral change interventions. This can be achieved through initiatives like parenting programs, which challenge and address unequal gender norms that place an excessive burden on girls within their own homes through household chores and in other households through domestic work (3, 24).

Some of the reviewed studies showed a surge in child labor in the agricultural sector since the start of the COVID-19 pandemic. The agricultural sector already accounts for the largest portion of child labor worldwide, with 70% of all children in child labor in agriculture (3). Working children in the agricultural sector face vulnerability to hazardous work through exposure to harmful substances, unsafe machinery, excessive physical burdens, and extremely long working hours (72). It is probable that families who have lost jobs elsewhere revert to subsistence farming as a means of survival. This may lead to a further rise in child labor in agriculture as children are compelled to work to support their families (61). Given the widespread occurrence of child labor in the agricultural sector, measures to combat child labor must be considered to reach rural communities. These measures should be accessible and adaptable to the conditions of the millions of children involved in agriculture globally (69).

The reviewed literature suggests that the economic impact of the COVID-19 pandemic is the primary cause for the increase of child labor during the pandemic. This impact has caused high levels of unemployment, greater poverty, lower remittances, and migration (3, 24). This results in children entering the workforce to meet their basic needs and support their families (3, 24). The number of children living in multidimensional poverty^3^ in 2021 is projected to increase by an additional 100 million children compared to 2019 (73). According to ILO and United Nations Children’s Fund, a 1% increase in poverty leads to at least a 0.7% increase in child labor (24). Unemployment of the parents due to economic shocks can lead children to enter to workforce to aid their families (24).

Some of the reviewed studies found that the demand for child labor is also likely to rise among employers as a source of cheap labor. The rise in the informal sector due to the COVID-19 pandemic may generate new demand for child labor, as the informal economy’s low skill requirements and the lack of regulation make it more conducive to employ children (24). In addition, employers prefer to employ children rather than adults because they are more obedient, easily controlled, and accept lower wages. Furthermore, employers demonstrate less concern regarding the violation of child labor laws due to the lack of effective enforcement of labor regulations, as many governments reduced or even removed labor inspections as a result of the COVID-19 precautions (24).

In addition, some of the reviewed studies found that a lack of access to food is a primary driver for children to enter the workforce. The COVID-19 pandemic has resulted in a dramatic increase in food insecurity, primarily due to public health restrictions, worsening economic conditions of families, and closure of schools (73). Food insecurity can force children into child labor as a means to earn money for purchasing food or to work in exchange for it. In addition, children who were already working are pushed to work longer hours or fall into more hazardous forms of labor to obtain food (3). UNICEF and the World Food Programme estimated that a total of 39 billion in-school meals have been missed during the first year of the pandemic in 2020 (74).

The reviewed studies also indicate that parental morbidity and mortality due to COVID-19 can also lead to a surge in child labor. The mortality or morbidity of parents or caregivers in poor countries will lead children to occupy part of their roles, such as domestic work and earning responsibilities. Various studies show that the death or sickness of parents can lead children to leave their education and enter child labor (75, 76).

The majority of the reviewed studies identify that temporary school closure caused by the response to the pandemic has also led to an increase in child labor. ILO estimates that more than one third of children in child labor were not enrolled in schools in 2020 (3). According to UNICEF, 24 million children who did not attend school during the pandemic will drop out for good (77). The closure of schools has also resulted in the interruption of supportive services which children rely on to survive like midday meals and school healthcare (43). Families may no longer be able to afford paying for their child’s schooling, leading children to permanently drop out of school (44).

### 4.2 Recommendations

Various measures to combat child labor during the COVID-19 pandemic were identified in the reviewed studies. To strengthen these measures, it is necessary to take actions aimed at identifying and reaching the most vulnerable children, including those who migrate seasonally with their families, or those who have been separated from their families due to various reasons. Targeted strategies and social welfare support are crucial for ensuring their well-being. Due to the multifaceted nature of the challenge, a multisectoral approach to ending child labor across various sectors such as education, social protection, business practices and child protection by governments, civil society, donors, and businesses is necessary.

The majority of the reviewed studies recommend that governments develop adequate social protection measures to help families experiencing poverty or vulnerable to living in poverty. ILO has recognized social protection measures, such as cash transfers, as a significant factor in the substantial reduction of child labor witnessed in the last 20 years (78). According to analysis from the ILO, an additional 8.9 million more children will likely engage in child labor without social protection mitigation measures (3). Globally, only 35% of children had access to social protection programs before the pandemic (79). Various factors such as transfer amounts, regularity and predictability, and duration of payments may be able to assess the impacts of social protection on child labor (80).

In addition to social protection, various child protection services are needed to prevent and remove children from child labor. Greater involvement of children, families and communities is necessary to strengthen child protection systems (61). Adequate community-based mechanisms can raise awareness of the negative impacts associated with child labor and promote positive parenting (61). The pandemic may have impacted the availability and ease of access to child protection services and other related resources for children, potentially rendering them susceptible to economic exploitation particularly in the informal sector (21). Therefore, social service workers should be adequately funded, and properly equipped and empowered to prevent and address child labor (24).

One recommendation identified in the reviewed studies includes financial support for families to allow children to continue their education. Evidence indicates that the bearing of negative economic turmoil on child labor can be lower in areas where schooling is more affordable (81). The removal of school fees has been shown to have a positive impact on child labor (82). Furthermore, the provision of school meals or take-home rations also proved to be effective in improving school attendance and reducing children’s work (83, 84). Another recommendation is for governments to improve education programs by providing greater funding to schools. Research shows that an improvement in school quality has been successful in reducing child labor (85).

A few of the reviewed articles identified the need for governments to strengthen laws and enforcement mechanisms in business operations and supply chains with the objective of preventing involvement in child labor or other human rights violations. Due diligence systems and responsible business practices have been debilitated by the pandemic, further endangering children’s rights (33). Even before the onset of the pandemic, research has shown the widespread prevalence of child labor in the global supply chain (86). Import restrictions on goods in critical supply chains can be relaxed as a result of global demand for medical supplies and equipment (87). In addition, businesses should fulfil their responsibility to apply laws and to apply appropriate measures to identify and prevent child labor in their operations and supply chains (3, 24).

Greater enforcement of child labor regulations was also identified as necessary to combat child labor during and following the pandemic. The diversions of financial and human resources as well as mobility restrictions due to the pandemic have limited workplace inspections (24). Various measures can be implemented to strengthen labor enforcement in such a setting. The backing of public labor inspectorates to detect and follow up on child labor cases, and the deeds of local community-based actors have proven effective, such as in Côte d’Ivoire and Ghana (33, 88). In addition, the use of new technologies, public-private partnerships, and workplace auditing schemes can support complying with the law (33).

Six of the reviewed studies also suggested that policymakers and civil society leaders focus on raising public awareness on the adverse impacts of child labor on health and development (20, 22, 36, 42, 57, 60). Stakeholders such as teachers and civil society leaders should be identified to help raise awareness about the COVID-19 pandemic (36). Research has shown that the impacts of economic factors on child labor is affected by cultural norms (71). The acceptance of some occupations as family occupations which should be upheld and passed along to their children strongly influenced parents’ decision to involve children in child labor (71).

### 4.3 Strengths and limitations of the current review

To the authors’ present knowledge, this is the first scoping review that aims to summarize the existing knowledge regarding the impacts of the COVID-19 pandemic on child labor, guide future research toward reversing the effects of the pandemic on child labor and inform policies to protect vulnerable children. The basis of this review was formed by a wide range of literature published throughout and after the COVID-19 pandemic, until January 21, 2022. This scoping review can provide an overview of existing research and can be used as an initial point to guide future policies needed to combat child labor following the COVID-19 pandemic, since the consequences of the pandemic will remain for some time to come. Like other scoping reviews, a limitation is the absence of quality assessment of the included studies. Finally, it was difficult to draw conclusions about changes in prevalence and trends of child labor due to the limited evidence available.

### 4.4 Suggestions for future research

The included studies were based primarily on literature reviews with few studies relying on primary data. None of the reviewed studies assessed health outcomes among working children or the relationship between household or child characteristics and child labor risk during the COVID-19 pandemic. Given the limited primary research identified, more quantitative longitudinal research on child labor is needed to measure occupational exposures, health outcomes and risk factors associated with child labor. Empirical research is needed to identify emerging patterns of child labor and develop effective intervention measures. Furthermore, the use of qualitative study design can provide a deep and complex understanding of the social, economic, and demographic context which influenced the decision of children or their families to join the workforce.

The included studies determined that emergency action should be implemented to prevent exploitation of children and to ensure that prior gains in eradicating child labor will not be lost. However, none of the reviewed studies assessed the effectiveness of these intervention measures during the pandemic at its peak. In addition, various child-specific and household-related characteristics such as poverty, neglect, vulnerability of children to various grades of violence, parental education status, gender, and household size among other factors can determine the effectiveness of social protection programs on the outcomes of child labor (89).

There is a scarcity of evidence regarding changes in hidden forms of child labor such as domestic work or children working in family businesses which are widely prevalent in developing countries (90). In addition, research is needed on the individual, community, and societal level risk factors of individual countries, which differ between geographic regions such as macroeconomic considerations in Latin America and the Caribbeans and violence in conflict-ridden. Environments (91). Furthermore, to better define the problem as industry-wide or as a function of industry context, research comparing similar industries employing child labor across different economic and geographical settings would be helpful.

We can conclude from the current review that there is limited empirical research on the effect of the COVID-19 pandemic on gender differences in occupational exposure and health outcomes among working children. Girls are particularly at risk of taking on additional domestic work or home care, which limit their educational opportunities, and are likely to be more exposed to work-related injuries (10). To devise the needed interventions and policies, and effectively mitigate adverse health outcomes, it is crucial to have a comprehensive understanding of the gender-related differences in risks and exposures. There is also a scarcity of research on working children from marginalized groups including migrants, refugees, internally displaced persons, or those belonging to minority groups. Therefore, future research should apply gender and diversity analysis to meet the specific needs of male and female working children. In addition, studies should also include an analysis of children belonging to marginalized communities, who are often more vulnerable to exploitation and violence. Developing strategies to target the most vulnerable working children is necessary. This is equally applicable to high-risk groups, such as the most vulnerable girls and young women, migrant and refugee children, and children in the poorest households.

## 5 Conclusion

The development, welfare, and health of children is significantly obstructed by child labor. At the beginning of 2020, an estimated 160 million children, 1 in 10 worldwide, were subjected to child labor (61). It is predicted that the effects of the COVID-19 pandemic may expedite the growth of child labor around the globe. In this scoping review, various factors that increase the risk of child labor were identified including increasing poverty, temporary school closure, loss of parents or caregivers to COVID-19, and greater demand for child labor among employers. Additionally, results of research to date, as summarized in this review, require governments and policymakers to develop social protection measures and educational interventions, as well as strengthen labor laws and enforcement mechanisms. In addition, civil society, and donors such as international agencies should work toward raising awareness on child labor and providing technical and financial support to various programs. Given the limited primary research identified, more field research on child labor is needed to detect new and emerging patterns of child labor and to develop effective intervention measures. Given the long-term impacts of the COVID-19 pandemic, there is also a need for further empirical research on the consequences of the COVID-19 pandemic on occupational exposure and health outcomes among working children as affected by gender differences and as part of a marginalized groups such as migrants, refugees, or minority groups. Additionally, both field research on child labor during the COVID-19 pandemic and further empirical research on the consequences of the pandemic, can better inform policymakers, government officials, and relevant stakeholders to better prepare and set protection measures for children during and after pandemics which may occur in the future.

## Data availability statement

The original contributions presented in the study are included in the article/Supplementary material, further inquiries can be directed to the corresponding author.

## Author contributions

RH: conceptualization. LH: database search strategy. MK, DH, and RH: literature review, screening articles, drafting early drafts, and the manuscript. MK, RH, DH, and RK: revising the final draft of the manuscript. All authors contributed to the article and approved the submitted version.

## Footnotes

^1^The 45 articles used in the scoping review are 3, 19-22, 24, 26-64.

^2^Global or regional studies are not included.

^3^The multidimensional poverty index measures “the percentage of households in a country deprived along three dimensions –monetary poverty, education, and basic infrastructure services – to capture a more complete picture of poverty” (﻿23﻿).
