# Supplementary material for: Child labor and associated risk factors in the wake of the COVID-19 pandemic: a scoping review
Source: Front Public Health. 2024 Feb 1;11:1240988. doi: 10.3389/fpubh.2023.1240988 (PMC10867312; doi:10.3389/fpubh.2023.1240988)
Supplement: Supplementary file 1 [file Table_1.DOCX]

Supplementary Material

Child Labor and Associated Risk Factors in the wake of the COVID-19 Pandemic: A Scoping Review

**Rima R. Habib^1*^, Moussa El Khayat^1^, Joly Ghanawi^1^, Reem S. Katrib^1^, Layal Hneiny^2^, Dana A. Halwani^1^**

^1^Department of Environmental Health, Faculty of Health Sciences, American University of Beirut, Beirut, Lebanon

^2^Wegner Health Sciences Library, University of South Dakota, Sioux Fall, South Dakota, United States

*** Correspondence:**Rima R. Habib

Faculty of Health Sciences

American University of Beirut
rima.habib@aub.edu.lb

Table S1: Database Search Strategies used for the Scoping Review

| **Databases** | **Strategy** | **Results** |
| --- | --- | --- |
| Medline – 1946 to January 21, 2022 | 1 coronaviridae/ or exp coronavirus/ or exp Coronavirus Infections/ or exp Betacoronavirus/  2 (Betacoronavirus or Beta-coronavirus or Coronavirus* or COVID).mp.  3 1 or 2  4 limit 3 to ez="20191101-20220124"  5 (("2019" adj (novel or new) adj corona*) or ("2019" adj (CoV or nCoV)) or (coronavirus adj (disease adj "2019")) or COVID19 or COVID-19 or ((Novel or New) adj Corona*) or SARS2 or SARS-CoV-2 or (SARS adj2 (coronaviridae or coronavirus)) or ((sars or Coronavirus) adj "2") or nCov or 2019ncov).mp.  6 COVID-19/ or COVID-19 Vaccines/ or exp COVID-19 Testing/ or SARS-CoV-2/  7 4 or 5 or 6  8 child labor/ or (child* adj3 (work* or labo?r or wage)).mp.  9 Social Problems/ or Employment/ or exp Work/ or (work* or labo?r or employ* or (social adj1 (problem? or issue? or dilema?))).mp.  10 Poverty Areas/ or Poverty/  11 Student Dropouts/  12 socioeconomic factors/ or economic factors/ or economic status/  13 rural population/ or suburban population/ or urban population/  14 Farms/  15 Farmers/  16 (((rural or urban or sub-urban or suburb*) adj2 (communit* or population? or societ* or place? or area? or cities or city)) or farm* or socio-economic* or socioeconomic or "economic status" or poor or povert* or (student* adj2 drop*)).mp.  17 10 or 11 or 12 or 13 or 14 or 15 or 16  18 exp child/ or (child* or kid? or boy* or girl* or minor? or teen? or adolescen*).mp.  19 9 and 17 and 18  20 8 or 19  21 7 and 20 | 694 |
| Scopus –1788 to January 21, 2022 | #1 TITLE-ABS-KEY ( ( ( "2019" W/1 ( novel OR new ) W/1 corona* ) OR ( "2019" W/1 ( cov OR ncov ) ) OR ( coronavirus W/1 ( disease W/1 "2019" ) ) OR covid19 OR covid-19 OR ( ( novel OR new ) W/1 corona* ) OR sars2 OR sars-cov-2 OR ( sars W/3 ( coronaviridae OR coronavirus ) ) OR ( ( sars OR coronavirus ) W/1 "2" ) OR ncov OR 2019ncov ) )  #2 TITLE-ABS-KEY ( ( ( ( rural OR urban OR sub-urban OR suburb* ) W/2 ( communit* OR population* OR societ* OR place* OR area* OR cities OR city ) ) OR farm* OR socio-economic* OR socioeconomic OR "economic status" OR poor OR povert* OR ( student* W/2 drop* ) ) )  #3 TITLE-ABS-KEY ( ( child* OR kid* OR boy* OR girl* OR minor* OR teen* OR adolescen* ) )  #4 TITLE-ABS-KEY ( ( work* OR labo*r OR employ* OR ( social W/2 ( problem* OR issue* OR dilema* ) ) ) )  #5 TITLE-ABS-KEY ( ( child* W/2 ( work* OR labo*r OR wage ) ) )  #6 (#1 AND ((#2 AND #3 AND #4) OR #5) | 1255 |
| Web of Science- 1900 to January 21, 2022 | (("2019" NEAR/1 (novel OR new) NEAR/1 corona*) OR ("2019" NEAR/1 (cov OR ncov)) OR (coronavirus NEAR/1 (disease NEAR/1 "2019")) OR covid19 OR covid-19 OR ((novel OR new) NEAR/1 corona*) OR sarsa OR sars-cov-2 OR (sars NEAR/3 (coronaviridae OR coronavirus)) OR ((sars OR coronavirus) NEAR/1 "2") OR ncov OR 2019ncovr) AND ((((rural OR urban OR sub-urban OR suburb*) NEAR/2 (communit* OR population* OR societ* OR place* OR area* OR cities OR city)) OR farm* OR socio-economic* OR socioeconomic OR "economic status" OR poor OR povert* OR (student* NEAR/2 drop*)) AND (child* OR kid$ OR boy* OR girl* OR minor* OR teen* OR adolescen*) AND (work* OR labo$r OR employ* OR (social NEAR/2 (problem* OR issue* OR dilema*))) OR (child* NEAR/2 (work* OR labo$r OR wage))) (Title) or (("2019" NEAR/1 (novel OR new) NEAR/1 corona*) OR ("2019" NEAR/1 (cov OR ncov)) OR (coronavirus NEAR/1 (disease NEAR/1 "2019")) OR covid19 OR covid-19 OR ((novel OR new) NEAR/1 corona*) OR sarsa OR sars-cov-2 OR (sars NEAR/3 (coronaviridae OR coronavirus)) OR ((sars OR coronavirus) NEAR/1 "2") OR ncov OR 2019ncovr) AND ((((rural OR urban OR sub-urban OR suburb*) NEAR/2 (communit* OR population* OR societ* OR place* OR area* OR cities OR city)) OR farm* OR socio-economic* OR socioeconomic OR "economic status" OR poor OR povert* OR (student* NEAR/2 drop*)) AND (child* OR kid$ OR boy* OR girl* OR minor* OR teen* OR adolescen*) AND (work* OR labo$r OR employ* OR (social NEAR/2 (problem* OR issue* OR dilema*))) OR (child* NEAR/2 (work* OR labo$r OR wage))) (Abstract) or (("2019" NEAR/1 (novel OR new) NEAR/1 corona*) OR ("2019" NEAR/1 (cov OR ncov)) OR (coronavirus NEAR/1 (disease NEAR/1 "2019")) OR covid19 OR covid-19 OR ((novel OR new) NEAR/1 corona*) OR sarsa OR sars-cov-2 OR (sars NEAR/3 (coronaviridae OR coronavirus)) OR ((sars OR coronavirus) NEAR/1 "2") OR ncov OR 2019ncovr) AND ((((rural OR urban OR sub-urban OR suburb*) NEAR/2 (communit* OR population* OR societ* OR place* OR area* OR cities OR city)) OR farm* OR socio-economic* OR socioeconomic OR "economic status" OR poor OR povert* OR (student* NEAR/2 drop*)) AND (child* OR kid$ OR boy* OR girl* OR minor* OR teen* OR adolescen*) AND (work* OR labo$r OR employ* OR (social NEAR/2 (problem* OR issue* OR dilema*))) OR (child* NEAR/2 (work* OR labo$r OR wage))) (Author Keywords) or (("2019" NEAR/1 (novel OR new) NEAR/1 corona*) OR ("2019" NEAR/1 (cov OR ncov)) OR (coronavirus NEAR/1 (disease NEAR/1 "2019")) OR covid19 OR covid-19 OR ((novel OR new) NEAR/1 corona*) OR sarsa OR sars-cov-2 OR (sars NEAR/3 (coronaviridae OR coronavirus)) OR ((sars OR coronavirus) NEAR/1 "2") OR ncov OR 2019ncovr) AND ((((rural OR urban OR sub-urban OR suburb*) NEAR/2 (communit* OR population* OR societ* OR place* OR area* OR cities OR city)) OR farm* OR socio-economic* OR socioeconomic OR "economic status" OR poor OR povert* OR (student* NEAR/2 drop*)) AND (child* OR kid$ OR boy* OR girl* OR minor* OR teen* OR adolescen*) AND (work* OR labo$r OR employ* OR (social NEAR/2 (problem* OR issue* OR dilema*))) OR (child* NEAR/2 (work* OR labo$r OR wage))) (Keyword Plus ®) or (("2019" NEAR/1 (novel OR new) NEAR/1 corona*) OR ("2019" NEAR/1 (cov OR ncov)) OR (coronavirus NEAR/1 (disease NEAR/1 "2019")) OR covid19 OR covid-19 OR ((novel OR new) NEAR/1 corona*) OR sarsa OR sars-cov-2 OR (sars NEAR/3 (coronaviridae OR coronavirus)) OR ((sars OR coronavirus) NEAR/1 "2") OR ncov OR 2019ncovr) AND ((((rural OR urban OR sub-urban OR suburb*) NEAR/2 (communit* OR population* OR societ* OR place* OR area* OR cities OR city)) OR farm* OR socio-economic* OR socioeconomic OR "economic status" OR poor OR povert* OR (student* NEAR/2 drop*)) AND (child* OR kid$ OR boy* OR girl* OR minor* OR teen* OR adolescen*) AND (work* OR labo$r OR employ* OR (social NEAR/2 (problem* OR issue* OR dilema*))) OR (child* NEAR/2 (work* OR labo$r OR wage))) (Topic) | 693 |
| CINAHL –1937 to January 21, 2022 | S1 (MH "Pneumonia, Viral") OR (MH "Pneumonia") OR (MH "Viruses+")  S2 (MH "Disease Outbreaks") OR (MH "Epidemiology+") OR MW Epidemiology  S3 S1 AND S2  S4 (MH "Coronaviridae+") OR (MH "Coronaviridae Infections+") OR TI ( Betacoronavirus or Beta-coronavirus or Coronavir* or corona-vir* or COVID ) OR AB ( Betacoronavirus or Beta-coronavirus or Coronavir* or corona-vir* or COVID ) OR MW ( Betacoronavirus or Beta-coronavirus or Coronavir* or corona-vir* or COVID )  S5 S3 OR S4  S6 TI ( (("2019" N2 (coronavirus or CoV or nCoV)) or COVID19 or COVID-19 or ((Novel or New or "2") N1 (corona-vir* or Coronavir*)) or SARS2 or SARS-CoV-2 or (SARS N1 ("2" or coronaviri* or corona-vir*)) or nCov or 2019ncov or ncov2019) ) OR AB ( (("2019" N2 (coronavirus or CoV or nCoV)) or COVID19 or COVID-19 or ((Novel or New or "2") N1 (corona-vir* or Coronavir*)) or SARS2 or SARS-CoV-2 or (SARS N1 ("2" or coronaviri* or corona-vir*)) or nCov or 2019ncov or ncov2019) ) OR MW ( (("2019" N2 (coronavirus or CoV or nCoV)) or COVID19 or COVID-19 or ((Novel or New or "2") N1 (corona-vir* or Coronavir*)) or SARS2 or SARS-CoV-2 or (SARS N1 ("2" or coronaviri* or corona-vir*)) or nCov or 2019ncov or ncov2019) )  S7 (MH "SARS-CoV-2") OR (MH "COVID-19 Pandemic") OR (MH "COVID-19") OR (MH "COVID-19 Testing") OR (MH "COVID-19 Vaccines")  S8 S5 OR S6 OR S7  S9 TI ( (work* OR labo#r OR employ* OR wage OR (social W1 (problem# OR issue# or dilema#))) ) OR AB ( (work* OR labo#r OR employ* OR wage OR (social W1 (problem# OR issue# or dilema#))) ) OR MW ( (work* OR labo#r OR employ* OR wage OR (social W1 (problem# OR issue# or dilema#))) )  S10 (MH "Social Problems") OR (MH "Employment") OR (MH "Work")  S11 S9 OR S10  S12 (MH "Farmworkers") OR (MH "Rural Population") OR (MH "Suburban Population") OR (MH "Urban Population") OR (MH "Economic Status") OR (MH "Economic Factors") OR (MH "Socioeconomic Factors") OR (MH "Student Dropouts") OR (MH "Poverty") OR (MH "Poverty Areas")  S13 TI ( (((rural OR urban OR sub-urban OR suburb*) N3 (communit* OR population# OR societ* OR place# OR area# OR cities OR city)) OR farm* OR socio-economic* OR socioeconomic OR "economic status" OR poor OR povert* OR (student* N3 drop*)) ) OR AB ( (((rural OR urban OR sub-urban OR suburb*) N3 (communit* OR population# OR societ* OR place# OR area# OR cities OR city)) OR farm* OR socio-economic* OR socioeconomic OR "economic status" OR poor OR povert* OR (student* N3 drop*)) ) OR MW ( (((rural OR urban OR sub-urban OR suburb*) N3 (communit* OR population# OR societ* OR place# OR area# OR cities OR city)) OR farm* OR socio-economic* OR socioeconomic OR "economic status" OR poor OR povert* OR (student* N3 drop*)) )  S14 S12 OR S13  S15 (MH "Child+")  S16 TI ( child* OR kid# OR boy* OR girl* OR minor# OR teen# OR adolescen ) OR AB ( child* OR kid# OR boy* OR girl* OR minor# OR teen# OR adolescen ) OR MW ( child* OR kid# OR boy* OR girl* OR minor# OR teen# OR adolescen )  S17 S15 OR S16  S18 S8 AND S11 AND S14 AND S17 | 169 |
| EMBASE-1947 to January 21, 2022 | #1 ('pneumonia'/de OR 'virus pneumonia'/de OR 'virus'/exp) AND ('epidemic'/exp OR 'epidemiology'/exp OR epidemiology:lnk)  #2 'coronaviridae'/exp OR 'coronavirus infection'/exp OR 'betacoronavirus'/exp  #3 betacoronavirus:ti,ab,kw OR 'beta coronavirus':ti,ab,kw OR coronavirus*:ti,ab,kw OR covid:ti,ab,kw  #4 #1 OR #2 OR #3  #5 #4 AND [1-11-2019]/sd  #6 ((2019 NEXT/0 novel):ti,ab,kw) OR ((2019 NEXT/0 cov):ti,ab,kw) OR ((coronavirus NEXT/0 disease NEXT/0 2019):ti,ab,kw) OR covid19:ti,ab,kw OR 'covid 19':ti,ab,kw OR (((novel OR new) NEXT/0 corona*):ti,ab,kw) OR sars2:ti,ab,kw OR 'sars cov 2':ti,ab,kw OR ((sars NEAR/2 coronaviridae):ti,ab,kw) OR coronavirus:ti,ab,kw OR sars:ti,ab,kw OR ((coronavirus NEXT/0 '2'):ti,ab,kw) OR ncov:ti,ab,kw OR 2019ncov:ti,ab,kw  #7 'coronavirus disease 2019'/exp OR 'severe acute respiratory syndrome coronavirus 2'/exp OR 'sars-cov-2 vaccine'/exp  #8 #5 OR #6 OR #7  #9 'child labor'/de OR ((child* NEAR/3 (work* OR labo$r OR wage)):ti,ab,kw)  #10 'social problem'/de OR 'employment'/de OR 'work'/exp OR work* OR labo$r OR employ* OR (social NEAR/1 (problem$ OR issue$ OR dilema$))  #11 'poverty'/exp OR 'school dropout'/de OR 'socioeconomics'/de OR 'economic aspect'/de OR 'economic status'/de OR 'rural population'/de OR 'urban population'/de OR 'suburban population'/de OR 'agricultural worker'/de OR 'farmers market'/de OR 'agricultural land'/exp OR (((rural OR urban OR 'sub urban' OR suburb*) NEAR/2 (communit* OR population$ OR societ* OR place$ OR area$ OR cities OR city)):ti,ab,kw) OR farm*:ti,ab,kw OR 'socio economic*':ti,ab,kw OR socioeconomic:ti,ab,kw OR 'economic status':ti,ab,kw OR poor:ti,ab,kw OR povert*:ti,ab,kw OR ((student* NEAR/2 drop*):ti,ab,kw)  #12 'child'/exp OR child*:ti,ab,kw OR kid$:ti,ab,kw OR boy*:ti,ab,kw OR girl*:ti,ab,kw OR minor$:ti,ab,kw OR teen$:ti,ab,kw OR adolescen*:ti,ab,kw  #13 #10 AND #11 AND #12  #14 #9 OR #13  #15 #8 AND #14 | 910 |
| Global Health –1973 to January 21, 2022 | title:(("Covid-19" OR coronavirus OR corona OR SARS-CoV-2 OR SARS2 OR "SARS 2 ") AND (child* OR kid OR kids OR adolescen* OR boy* OR girl*) AND (labor OR labor OR labour OR work* OR wage OR employ*)) AND ab:(("Covid-19" OR coronavirus OR corona OR SARS-CoV-2 OR SARS2 OR "SARS 2 ") AND (child* OR kid OR kids OR adolescen* OR boy* OR girl*) AND (labor OR labor OR labour OR work* OR wage OR employ*)) AND subject:(("Covid-19" OR coronavirus OR corona OR SARS-CoV-2 OR SARS2 OR "SARS 2 ") AND (child* OR kid OR kids OR adolescen* OR boy* OR girl*) AND (labor OR labor OR labour OR work* OR wage OR employ*)) | 13 |
| **Total Results** |  | 3734 |

Table S3: Heat related health outcomes, risk factors and preventative measures extracted from the reviewed studies

| **Reference** | **Study population (sample size)** | **Gender/Age** | **Heat-related outcomes (only statistically significant in quantitative studies and all outcomes in qualitative studies)** | **Protective factor (only statistically significant in quantitative studies and all risk factors in qualitative studies)** | **Risk factor (only statistically significant in quantitative studies and all risk factors in qualitative studies)** | **Non-significant association** | **Prevalence of preventive measures** |
| --- | --- | --- | --- | --- | --- | --- | --- |
| Acrury et al. 2015 | Farmworkers (101)  Worked outside in extremely hot weather conditions: 68  Worked inside in extremely hot weather conditions: 18 | 101 males  Age range: 30-70 | - 35.6% of the of the total sample reported heat illness - 52.9% of the sample that worked outside in extremely hot weather conditions had at least one heat illness symptom   *HRI symptoms:*   - Sudden muscle cramps (outside extremely hot weather conditions 25% vs inside in extremely hot weather conditions 27.8%) - Nausea or vomiting (10.3% vs 22.2%) - Hot, dry skin (32.4% vs 61.1%) - Confusion (13.2% vs 22.2%) - Dizziness (16.2% vs 27.8%) |  |  |  | - Drinking more water (58.4% of total sample vs 86.8% working outside in extremely hot conditions) - Taking breaks in shaded areas (59.4% vs 88.2%) - Going to air-conditioned places (19.8% vs 29.4%) - Changing work hours (16.8 % vs 25%) - Changing work activities (13.9% vs 20.6%) - Changing hours or activities (19.8% vs 29.4%) - Changing hours and activities (10.9% vs 16.2%) |
| Acrury et al. 2019 | Latinx child  farmworkers (202) | 35 males  50 females  Age range: 10-17 | - 45.5% heat-related illness in the past year   *HRI symptoms reported:*   - 25.7% dizziness - 21.8% sudden muscle cramps - 17.3% hot, dry skin - 7.4% nausea or vomiting - 5.0% confusion - 1.5% fainting |  | *Association with greater odds of HRI:*   - Increased age (OR 1.41, 95% CI 1.18, 1.69) | • Gender  • Migrant status  • Work with adult relative  • Amount of work in last three months |  |
| Acrury et al. 2020 | Latinx child  farmworkers (202) | 76 females  126 males  Age range: 10-17 | - 45.5% of participants reported HRI |  | *Association with greater odds of HRI in bivariate models:*   - Pesticide safety training (OR 2.03, 95% CI 1.08, 3.84)   *Association with greater odds of HRI in multivariate models:*   - Oldest farmworkers aged 16-17 years (OR 4.52, 95% CI 1.93, 10.57) compared to youngest aged 10-13 years - Pesticide safety training (OR 3.26, 95% CI 1.39, 7.65) | *Bivariate models:*   - Appropriate work clothing - Safety training—tool use - Safety training—machinery - Work piece-rate - Field sanitation services - Safety/risk attitudes - Vulnerability - Work safety climate scale - Supervisor interested in doing the job fast and cheaply versus as much as possible - Supervisor doing as much as possible to do the job safety versus as much as possible   *Multivariate models:*   - Age (14–15 years versus 10–13 years) - Gender - Migrant worker - Work with adult relative - Amount of Work in Last Three Months Clothing - Safety training—tool use - Safety training—machinery - Safety/risk attitudes - Vulnerability - Work safety climate scale - Supervisor interested in doing the job fast and cheaply versus as much as possible - Supervisor doing as much as possible to do the job safety versus as much as possible |  |
| Alam Shah et al. 2021 | Farmworkers and gardeners (not specified) |  | - Sperm count, motility and morphology are much better compared to bakers and oven workers |  |  |  |  |
| Alemu Gelaye et al. 2021 | Farmworkers (950) | Mean age: 26  Male: 943  Female: 7 | - 60.9% had three or more HRI symptoms   *HRI symptoms*   - 45.1% skin rash - 21.4% painful muscle cramp/spasm - 25.8% Irritability - 40.4% Headache - 42.9% Profuse sweating - 45.1% Fatigue - 45.3% Dizziness - 56.8% Extreme weakness - 31.2% Blurred vision - 15.1% Fainting /unconscious - 47.5% Confusion /restlessness |  | *Association with injuries*   - Heat stress (AOR: 1.48, 95% CI: 1.09-1.98) |  |  |
| Arnold et al. 2020 | Latinx child farmworkers (165) | 106 males  59 females  Age range: 10-17 | - 47.8% of participants   reported at least one HRI symptom   - 22% experienced at least two symptoms   *HRI symptoms reported:*   - 29.1% dizziness - 21.8% sudden muscle cramps - 17.6% hot, dry skin - 8.5% nausea or vomiting - 6.1% confusion - 1.8% fainting | *Association with lower HRI:*   - Take extra breaks (62.0% vs. 39.7%, p= .0045) | *Association with higher HRI:*   - Older participants compared to younger (10-13, 60.8%; 14-15, 44.2%; 16-17, 23.5%, p= 0011) - Go to air-conditioned places during breaks or after work (59.6 % vs. 41.7 %,p= .0279) | - Gender - Farmworker status - Years of farm work experience - Work with older relative - Pay structure - Recipient of pay - Extra water - Breaks in shade - Hours changed - Tasks changed | - 90.9% drank extra water - 87.9 % took breaks in shaded areas - 55.8 % took extra breaks - 43% changed work hours - 34.6 % gone to air-conditioned places during breaks or after work - 10.9 % changed tasks - Some reported leaving work early |
| Bethel et al. 2014 | Farmworkers (100) | 60 males  40 females  Age range:  18-62 | - 30% of participants reported experiencing 2 or more HRI symptoms   *HRI symptoms reported:*   - 10% skin rash/skin bumps - 9% painful muscle cramps/spasms - 7% dizziness/light-headedness - 1% fainting - 24% headache - 50% heavy sweating - 14% extreme weakness/fatigue - 2% nausea/vomiting - 3% confusion - 36% none |  |  |  | - 48.3% gradually increased work hours at start of season - 73% drank water at least once per hour past week - 40% had no cooling measure at work   *Head protection usually worn at work:*   - Baseball cap (94%) - Wide brimmed hat (21.0%) - Other hat (2.0%) - Bandana (75.0%) - Hood from hooded sweatshirt (63.0%)   *Clothing usually worn at work*   - Light-colored short-sleeved shirt (9.0%) - Dark-colored short-sleeved shirt (2.0%) - Light-colored long-sleeved shirt (90.0%) - Dark-colored long-sleeved shirt (23.0%) - Shorts (4.0%) - Pants (97.0%) - Jacket (72.0%) |
| Bethel et al. 2017 | Farmworkers (197)  Oregon state: 100  Washington state: 97 | 60 males  40 females  Mean age:31.8  51 males  46 females  Mean age:40.4 |  |  |  |  | *Types of beverages consumed:*   - 98.5% water - 46.2% sports drink - 8.6% energy drink - 24.9% juice - 4.6% iced coffee or tea - 10.7% hot coffee or tea - 48.2% soda - 2% other drink - Shade structures (Oregon 26% vs. Washington 6%) - Rest stations (19% vs. 6%) - Trees (47% vs 91.8) - Fans (4% vs 2.1%) - Building with air conditioning (1% vs 0%) - Cars with air conditioning (14% vs. 3%) - Mister (3 % vs 0%) - Wet clothes (40% vs. 2.1%) - Hose (14% vs. 2%) - Jump in river or canal (1% vs 0%) - HRI training (54% vs 34%) - Gradual increase of work hours at start of season (48.3% vs 34.4%)   *Headwear usually worn at work:*   - Baseball cap (94% vs 76.3%) - Wide-brimmed hat (21% vs 22.7%) - Other hat (2% vs 0%) - Bandana (75% vs 25.8%) - Hood from hooded sweatshirt (63% vs 15.5%) |
| Biggs et al. 2011 | Forestry workers (182)  Autumn: 103  Winter:79 | 64 males  39 females  Mean range:  37  68 males  11 females  Mean range:26 | - Preshift dehydration was 43% in autumn and 47% in winter (USG > 1.020 g ml) - Significant increase (P= 0.001) in the post-shift dehydration as 64% (P = 0.001) in autumn and 63% (P=0.043) in winter - 21% in autumn and 23% in winter lost >2% of their body weight across the shift - 44% of all workers were dehydrated pre-shift(USG>1.021) - 63% of all workers were dehydrated post-shift (USG >1.020) |  |  |  |  |
| Bodin et al. 2016 | Sugarcane workers (116)  Intervention group (Inland): 56  Nonintervention group (Coastland): 60 | Intervention group:  55 males  1 female  Nonintervention group:  46 males  14 females  Age range: 18-63 | Compared to pre-intervention, in post-intervention workers reported reduction of:   - Exhaustion - Nausea - Cramps - Dry mouth - Low/dark urine - Fever - Dizziness - Disorientation - Fainting - Stomachache - Headache |  |  |  |  |
| Budhathoki et al. 2019 | Farmworkers (350) | 220 males  130 females  Age range: 38.72; 12.9 | - 37% experienced heat related health problems   *HRI symptoms reported:*   - 73% fatigue - 63% dizziness - 41% headache - 28% nausea - 24% confusion - 12% heat rash - 8.3% fainting - 8% loss of concentration - 2.3% heat strokes |  |  |  | - 96% wore broad brimmed hats or used umbrellas - 65% rescheduled their work shifts - 61% stopped their outdoor farm activities - 93% rested in the shade and slowed down their work pace - 54% drank more cold water, stayed in sheds, stayed inside the house, and used wet clothing - Bathed in cold water |
| Butler- Dawson et al. 2017 | Sugarcane workers (330) | Male: 330  Median age: 28 | - 14% at baseline had eGFR between 60 and 89 ml/min/1.73m2 and 1% < 60ml/min/1.73m2 - Cross-harvest decline in eGFR in 37% of sugarcane cutters, 31% with a decline in eGFR 1- 20% and 6% with a decline in eGFR > 20% - 3% of cutters had eGFR< 60 ml/min/1.73m2   *Decline in 0%ΔGFR:*   - Workers at Mill A had 12.03% decline in eGFR compared with workers at Mill B. - Local workers had 6.30% decline in eGFR compared to highland workers - Current smokers had 5.80% decline in eGFR compared to Never/Former smokers - Workers with pre-employment eGFR <90 had 7.31% improvement in eGFR compared with workers with an eGFR ≥ 90 |  | *Decline in kidney function (less than 0%ΔGFR):*   - Mill A (OR 2.60, 95% CI=1.39,4.80) - Local worker (OR: 2.15, 95% CI: 1.28,3.60) - Current smoker (OR: 2.33, 95% CI: 1.17,4.63)   *Severe decline (more than 20% decline) in %ΔGFR:*   - Pre-employment eGFR less than 90 (OR: 4.23, 95% CI: 1.12,15.99) - Local worker (OR: 4.37, 95% CI: 1.41,13.52) - Current smoker (OR: 5.27, 95% CI: 1.54,17.99)   *Mild decline (0% to 20% decline) in %ΔGFR:*   - Local worker (OR: 1.92, 95% CI: 1.12:3.30) - Working at Mill A (OR: 2.48, 95% CI: 1.30:4.72) |  |  |
| Butler-Dawson et al. 2019 | Sugarcane workers (517) | 517 males  Age range: >18 | - 81% of sugarcane workers had at least one AKI (indicated by increase in serum creatinine of 26.5 μmol/L or 50% or more from the pre-shift value) during the study period - AKI cumulative incidence over a work shift was 47% in February, 51% in March and 45% in April | *Association with lower AKI (univariate analysis):*   - Average WBGT (per 1 °C) (OR 0.90,95% CI 0.82, 0.98) - Maximum WBGT (per 1 °C) (OR 0.89 95% CI 0.82, 0.96) - More work shift hours (OR 0.89 95% CI 0.83, 0.95)   *Association with lower AKI (multivariate analysis):*   - Higher baseline eGFR (OR 0.98, 95% CI 0.97–0.99) - Higher electrolyte solution intake were associated (OR 0.94, 95% CI 0.89–0.99) | *Association with higher AKI (univariate analysis):*   - Age (OR 1.01, 95% CI 1.00–1.03) - Baseline eGFR (OR 0.98, 95% CI 0.98–0.99) - Pre-shift urinary-specific gravity (OR 1.41, 95%   CI 1.19–1.67)   - Post-shift specific gravity (OR 1.48; 95% CI 1.27–1.72) - Electrolyte solution intake (OR 0.89, 95% CI 0.85–0.93) - Rest breaks (OR 0.83, 95% CI 0.75–0.93) - Work shift hours (OR 0.89, 95% CI 0.83–0.95) - Job type (OR 3.10, 95% CI 2.29–4.19) - Average WBGT (OR   0.90, 95% CI 0.82–0.98   - Maximum WBGT (OR 0.89, 95% CI 0.82–0.96)   *Association with higher AKI (multivariate analysis):*   - Dehydration indicated by higher post-shift specific gravity (OR 1.24, 95% CI 1.02–1.52) - NSAIDs and increasing post shift specific gravity (OR 8.38, 95% CI 1.67–42.16) | *Univariate analysis:*   - Local home residence - Hypertension - BMI - Previous harvests - Body weight change - Specific gravity, percent change - Sugary beverage intake - Smoked cigarette - NSAID use - Alcohol intake   *Multivariate analysis:*   - Age - Hypertension - Work shift hours - Rest breaks - Pre-shift specific gravity - Average WBGT |  |
| Butler-Dawson et al. 2020 | Sugarcane Workers (105) | Mean age: 30  Male: 105 | - Average serum creatinine was 85.75 μmol/L - Average eGFR was 105.73mL/min/1.73 m2 - Average urine NGAL was 9.71 ng/mL |  | *Association with serum creatinine:*   - HbA1c (β:19.28; 95% CI: 6.73 to 31.84) - Age (β:0.43 95% CI: 0.01-0.84)   *Association with eGFR:*   - HbA1c (β: –15.59; 95% CI: –26.40 - –4.78) - Age (β: –1.13; 95% CI: –1.46 to –0.80)   *Association with urine NGAL:*   - Weight change, kg (β: 0.29 ; 95% CI: 0.01-0.57) |  |  |
| CDC, 2008 | A Hispanic worker | Male  Age: 56 | - Death due to heat stroke - Core body temperature was 42°C at time of death - The man developed confusion before death |  |  |  | - The worker had not received HRI training |
| Cortez et al. 2009 | Sugarcane workers (22) | Age not specified  Gender not specified |  |  |  |  | - Seven workers drank from 7 to 8 L as temperature increased - Although temperature increased to maximum values many workers did not follow the rehydration measures and drank less than 6 L which is considered low |
| Crowe et al. 2009 | Sugarcane field workers (sample size not specified) |  |  |  | - Temporary workers are reluctant to take breaks since they are paid by piece |  | - Most field workers take between 2 and 10 liters of water into the field with them - Most workers reported drinking either coffee or ‘fresco’ (fruit juice mixed with water and sugar) - Workers use long sleeves, long pants and neck covering |
| Crowe et al. 2015 | Sugarcane cutters (169)  Harvesters: 106  Median age: 34  Non-harvesters: 63  Median age: 37 | (gender not specified)  Median age: 34  Median age: 37 | - Heat and dehydration symptoms experienced at least once per week were significantly different between harvesters and non-harvesters (P<0.05) with the exception of vomiting and dry mouth   *HRI symptoms at least once per week:*   - Headache (harvesters 50.9% vs non-harvesters 25.4%) - Tachycardia (34.9% vs 4.8%) - Muscle cramps (24.5% vs 11.1%) - Fever (17.9% vs 3.2%) - Nausea (17% vs 0%) - Difficulty breathing (13.2% vs 0%) - Dizziness (11.3% vs 1.6%) - Swelling of hands/feet (7.5% vs 0%) - Vomiting (3.8% vs 0%) - Dry mouth (32.1% vs 22.2%) - Dysuria (28.3% vs 3.2%) - Heat and dehydration symptoms increased as heat exposure categories increased (office and various workers, field and plant workers and harvesters) |  |  |  |  |
| Culp et al. 2019 | Farmworkers (168)  Cross sectional (CS) group: 148  Intensive Surveillance (IS) Group: 20 | 168 males  Age range: 18-65 | *CS group:*   - Extreme thirst (21.9% among participants between the ages of 18-34 years 21.9% vs. 16.5% among participants equal or older than 35 years 16.5%) - Muscle cramps (7.8% vs 7.1%) - Confusion (4.7% vs 7.1%) - Light-headed or dizzy (4.7% vs 5.9%) - Nausea (4.7% vs 3.6%) - Chest pounding (3.1% vs 1.2%)   *Association with uncomfortable Physiological intensity (PI) score (0-5) PI among IS group:*   - Higher body temperature (F ratio = 16.41, p < .001) - Kilocalories per hour (F ratio=8.41, p=0.001)   *Association with uncomfortable WBGT among IS group:*   - Higher heart rate (F ratio: 4.59, p = 0.014) - Higher breathing rates (F ratio: 6.48, p = 0.003) - Higher PI scores (F ratio: 5.11, p = 0.003) |  |  |  | *CS group:*  *Types of beverages consumed:*   - 68.9% water - 19.6% soda - 4.7% sports drinks |
| Das et al. 2013a | Control and experimental group (170)  Experimental: Groundnut farmers (85)  Control: Office workers (85) | Gender not specified  Mean age: 31.9;6.82  Gender not specified  Mean age: 27.2;5.4 | *Rate at work vs. just after work*   - Heart rate:74.4 vs. 121.5 beats/min - Systolic blood pressure: 110.2 vs. 132.2 - Diastolic blood pressure: 71.9 vs. 80.1   *Rate among farmers vs. office workers*   - Maximum heart rate: 188.1 vs. 192.8 - Heart Rate Reserve: 113.8 vs. 117.7 - Net Cardiac Cost: 47.1 vs. 31.8 - Relative Cardiac Cost: 63.45 vs. 42.6 |  |  |  |  |
| Das et al. 2013b | Control and experimental group (240)  Experimental: Child agricultural workers (120)  Control (120) | 63 males  57 females  Age range: 10-16 | - Change of heart rate (workers 81.5 beat/min vs control 32.8 beat/min) - Change of systolic blood pressure (35 mmHg vs 19.3 mmHg) - Change of diastolic blood pressure (7 mmHg vs 4.3 mmHg) |  |  |  |  |
| Diniz et al. 2021 | Sugarcane Cutters (17) | Mean Age: 26  Male: 17 | - No participants had abnormal eGFR_Scr-Scy_ (eGFR calculated using both serum creatinine and cystatin C) - 2 participants had low eGFRScr (eGFR calculated using serum creatinine) - 7 participants had low Cystatin C while none had high Cystatin C |  |  |  |  |
| Ekiti et al. 2018 | Sugarcane workers (204)  Office: 23  Factory:57  Field:124 | 153 males  51 females  Mean age: 38.8;9.8 | - 3.4% of participants had CKD (indicated by proteinuria and/or GFR< 60 ml/min/1.73 m2 persistent after 3 months) - CKD prevalence was 7% in factory workers compared to 0% and 2.4% in office and field workers - 2.9% had persistent proteinuria which was mild - 0.5% had GFR < 60 ml/min/1.73 m2 |  | *Association with higher CKD:*   - Age ≥ 40 years (OR = 18.7, 95% CI = 1.5–236.4, p = 0.024) | - Sex - Contract type - Duration of employment - Socio-economic status - Exposure to agrochemicals, heavy metals and heat - Alcohol use - Chronic use of herbal medicines - Family history of CKD - Obesity and overweight - NSAID use - Tobacco use - Hypertension - Diabetes |  |
| Fitria et al. 2020 | Rice harvesters (354) | Male: 354  Age: 20-65 | - The overall prevalence of CKD was 24.9% while CKDu was 18.6%. |  | *Association with CKDu:*   - Farm location (high altitude versus low altitude location) (Prevalence Odds Ratio (POR): 2.0; 95% CI: 1.2–3.5) |  |  |
| Fleischer et al. 2013 | Farmworkers (405) | 326 males  79 females  Age range: >18 | - 1/3 reported experienced three or more symptoms in the past week - 71% experienced at least one symptom in the past week   *HRI symptoms:*   - 50.8% headache - 44.9% hot, dry skin - 33.7% sudden muscle cramps - 24.6% dizziness - 16.7% nausea or vomiting - 15.5% confusion - 4.4% fainting | *Association with a reduction in prevalence of three or more HRI symptoms:*   - Increasing breaks in the shade reduces (9.2%) - Increasing access to medical attention (7.3%) - Reducing soda intake by (6.7%) - Increasing access to regular breaks (6.0%) |  | - Go cool down - Sports drinks - Water - Time in Georgia this year - Education - Access to shade - Load/pack outside - Alcohol - Change work duties - Work days/week - Juice - Wear sunscreen - Work hours/day | *Drink more of beverage during hot conditions:*   - 95.3% water - 83.8% sports drinks - 62.6% juice - 53.5% soda - 21.6% energy drinks - 6.8% coffee or tea - 6.5% alcohol |
| Flocks et al. 2013 | Fernery and nursery workers (35) | 35 females  Age range: 18-55 | - Prevalence of general health effects include headaches, dizziness/fainting, respiratory problems, vomiting, and exacerbated high or low blood pressure |  |  |  |  |
| Frimpong et al. 2013 | Farmworkers (308) | Gender not specified  Age not specified | - Prickly heat (17% male vs 17% female) - Heat cramp (29% vs 17%) - Heat exhaustion (40% vs 23%) - Malaria (77% vs 54%) - Cerebro-spinal meningitis (23% vs 10%) |  |  |  |  |
| Frimpong et al. 2020 | Farmworkers (308) | Males: 186  Females: 122  Age not specified |  |  |  |  | - 26.6% got away to a shade for a while - 4.2% removed clothing for free air - 19.8% drank water regularly - 16.9% wore a hat - 32.5% wore of airy dress - 3.2% covered head with traditional scarf - 10.4% wore traditional cloth which is airy - 46.1% ate traditional food which induces regular intake of water - 40.3% didn’t eat traditional food |
| García-Trabanino et al. 2015 | Sugarcane cutters (189) | 168 males  21 females  Age range: 18-49 | - 20% of the men had a pre-shift serum creatinine level above 1.2 mg/dL - 27% of the men had a serum uric acid level above 7.0 mg/dL. - 14% had a pre-shift GFR less than60 mL/min/1.73 m^2^ - 3 workers had severely reduced eGFR (<30 mL/min) - 10% increase in serum creatinine, urea nitrogen, and uric acid during post-shift as compared to pre-shift - Mean urine specific gravity was 1.016 pre-shift and 1.020 post-shift - Increase of mean urinary creatinine from 1.1 to 1.9 g/L - 1/3 of workers lost >0.5 kg of body weight | *Association with increased GFR linear regression models:*   - Liquid intake (0.06 mg/dl decrease in serum creatinine for each dL per hour of liquid intake, P=0.07)   *Association with increase post shift body weight*   - One extra liter of fluid increased post-shift body weight with about 0.5 kg (P=0.008) | *Association with reduced GFR among* logistic regression model*:*   - Age (OR 1.09 per year, 95% CI 1.02–1.16, P=0.008) - Region – coastal region (where temperature and humidity is higher) versus the other two regions combined (OR 3.5, 95% CI 1.3–9.4, P=0.01) - Association with ever use of carbamate insecticide when replacing any use of pesticides   *Association with increased post-shift serum creatinine in multiple linear regression models:*   - WBGT (about 2% increase in serum creatinine per degree of WBGT, p=0.001) | *Logistic regression model:*   - BMI - Smoking - Kidney stones - Hypertension - NSAIDS use - Diuretics - Number of previous harvests - Any use of pesticides   *Multiple linear regression:*   - Work time - Region | - The mean liquid intake was 0.3 L at breakfast and 3.3 L during work (mean 0.8 L per hour) - 90% was water |
| Glaser et al. 2020 | Sugarcane workers  Harvest 1 (525)  Harvest 2 (567) | Mean age Harvest 2:  Field support staff (FS)  31 (7)  Irrigation repair workers (IR)  29 (7)  Seed cutters (SC): 28 (7)  Burned cane workers (BCC): 31 (8) | *After intervention is introduced:*   - Lower decline in cross harvest eGFR among BCC (6, 95% CI 2 to 9) - Lower decline in cross harvest eGFR among SC (2 −1, 4) - IKI (indicated by increase in serum creatinine by ≥0.30 mg/dL or ≥1.5 times increase from baseline to end-harvest) was 70% (95% CI 90% to 50%) lower among BCC - Seed cutter groups with the worst compliance had the most cases of IKI whereas the groups with the best compliance had only one case |  |  |  | - Workers reported larger water and boli (300 mL electrolyte solution bags) intake after intervention is introduced |
| Grimbuhler & Viel et al. 2021 | Vineyard Workers (30) | Age range: 21-59  Female: 19  Male: 11 | - Skin temperature at rest (°C) (mean 33.9 vs minimum 31.7 vs maximum 35.8) - Mean skin temperature during work (°C) (34.9 vs 32.34 vs 36.1) - Mean heart rate (bpm) (113.5 vs 78.7 vs 165.3) - Net cardiac cost (bpm) (24.1 vs 7.1 vs 62.7) - Relative cardiac cost (%) (23.1 vs 10.1 vs 87.2) - Cardiac workload score (unitless) (8.8 vs 2.0 vs 15.0) |  | *Association with skin temperature*  *at rest (°C):*   - BMI (β: 0.114, 95% CI: 0.020, 0.208)   *Association with mean skin*  *temperature during work (°C):*   - Male gender (β: 1.447; 95% CI: 0.922-1.972) - Experience in vine-lifting (β: 0.038; 95% CI: 0.009-0.067)   *Association with relative cardiac cost (%):*   - Task duration (β: 0.219; 95% CI: 0.033-0.405) - Dry-bulb temperature (°C) (β: 3.652; 95% CI: 0.888-6.416)   *Association with Net cardiac cost (bpm):*   - Experience in vine-lifting (β: −0.476 95% CI: −1.001-0.049)   *Association with Mean heart rate (bpm):*   - Dry-bulb temperature (°C) (β: 4.084; 95% CI: 0.442-7.726)   *Association with cardiac workload score:*   - Dry-bulb temperature (°C) (β: 0.644; 95% CI: −0.046-1.334) | - Age was not associated with any physiological parameters |  |
| Gun, 1995 | Sheep Shearers (43) | 43 males  Age range: 18-59 | - Daily average sweat loss was 6.1 Kg - Average dehydration was 2·8% of body mas - Drink was 72% of sweat loss - Average rectal temperature was 37.7°C - Afternoon mean values of rectal temperature exceeded 38·0°C in 4 of the l5 observations made when WBGT > TLV | *Association with decreased rectal temperature:*   - Heavier alcohol consumption was associated with 0·16°C lower (p = 0·02) rectal temperature | *Association with increased rectal temperature:*   - WBGT (Percentage of variation explained 27%, partial regression Coefficients 0·029) - Vapor pressure (16%, 0.054) - Fat free mass (4%, 0.008) - Alcohol (5%, -0.001)   *Association with sweat loss:*   - WBGT (61%, 508) - Fat free mass (16%, 116) | - Added radiant heat - Thermal environment - Body fat content | - Drinking water |
| Hamerezaee et al. 2021 | Farmworkers (87) | Mean age: 37.10  Male: 87 | - Mean systolic blood pressure was 127.24 ±14.12 mmHg - Mean diastolic blood pressure was 76.20±10.37 mmHg - Mean heart rate 75.71±12.15 was beats/min - Mean oral temperature was 36.01±0.64 °C - Mean skin temperature was 34.43±1.67 °C |  |  |  |  |
| Hansen et al. 2020 | Case study of seasonal migrant workers |  |  |  | - Cultural and language barriers to understanding safety training and instructions - Lack of ability to make autonomous decisions about their health and safety - Piece rate work encourages workers to continue working |  |  |
| Hansson et al. 2019 | Sugarcane workers (525)  Field support (low workload): 52  Irrigation workers  (moderate):128  Seed cutters (high):188  Cane cutters: (very high):157 | 394 males  131 females  Age range: 18-60 | *IKI prevalence:*   - 27% in burned sugarcane cutters - 9% in seed cutters - 2% in both field support staff and irrigation workers - eGFR decreased by 4.6 (95% CI 3.2 to 6.0) mL/ min/1.73 m2 during harvest - Of the 427 workers examined at baseline and end-harvest, 32 (7%) had IKI | *Association with lower eGFR and IKI:*   - 1-year age increase associated with a 0.96 (95% CI 0.92 to 0.99) IR decrease | *High-moderate workload groups had higher IKI:*   - Burned cane cutters (Incidence ratio 9.3, 95% CI 3.2 to 28.3) - Seed cutters (3.3, 95% CI 1.1 to 9.9)   *Association between workload and cross harvest GFR:*   - Irrigation repair and field support (mean 0; 95% CI -2,1) - Seed cutters ( -5, -7, -3) - Cane cutters (-9, -13, -6) | - Sex - Pesticide use - NSAID use - Smoking - Change in 24-hour liquid intake over harvest - Sugary beverage intake per 24 hours at end of harvest - Diabetes - Hypertension |  |
| Hansson et al. 2020 | Male Sugarcane cutters (>800) | Age range: >18 |  | *Association with reduced risk of IKI (indicated by ≥0.3 mg/dL or ≥50% increase in serum creatinine since baseline):*   - Morning boli intake (300 mL electrolyte sachets) (Incidence Ratio IR 0.5, 95% CI 0.2–0.9) | *Association with increased risk of IKI:*   - Sugary drink intake greater than 1L (IR 4.4, 95% CI 1.0–19) - NSAID use at least once per week (IR 2.1, 95% CI 1.2–3.8) | - Age |  |
| How et al. 2020 | Farmworkers (58)  Agroecology (no pesticide use): 33  Conventional rice farmers (pesticide users): 25 | 58 males  Age range: 20-60 | - Significant difference in blood pressure and blood glucose (p < 0.05) among organic and conventional farmers - Significantly higher HSI among conventional rice farmers (p < 0.001)   *Compared to agroecology farmers, conventional farmers have higher odds of:*   - Prehypertension (OR: 3.89, CI: 0.904-16.72) - Hypertension (OR: 15.0, CI: 3.153-71.367) - Prediabetes (OR: 13.75, CI: 2.86-66.03) - Diabetes (OR: 5.56, CI: 1.37–22.56) |  |  |  |  |
| How et al. 2021 | Agroecology farmworkers (91)  Conventional farmworkers (104) | Age range: 18-67  Male: 60  Female: 31  Age range: 25-76  Male: 78  Female: 26 | - The minimum and maximum number of HRI symptoms was 4 and 8 respectively among agroecology farmworkers - The minimum and maximum number of HRI symptoms was 1 and 6 respectively among conventional farmworkers |  |  |  |  |
| Ioannou et al. 2021 | *Observational studies*  Agricultural workers (36)  *Interventional studies*  Agricultural workers (Cyprus) (6)  Agricultural workers (Qatar) (34) |  | *Intervention studies (Cyprus)*   - Use of mechanical fruit cart and improved work/rest ratio did not lower core temperature, skin temperature and heart rate - Use of ventilated garments reduced skin temperature (p = 0.009; d = -1.59) without impacting core temperature and heart rate   *Intervention studies (Qatar)*   - Hydration strategy increased heart rate (p = 0.009; d = 0.69) without affecting core temperature and skin temperature - Use of evaporative garments had no effect on core temperature, skin temperature and heart rate - Improved breaks increased heart rate (p = 0.006; d = 0.84) without affecting core temperature and skin temperature |  | *Association with skin temperature:*   - WBGT (R2 = 0.941; F(1,10) = 159.098, p < 0.001) | - No association was identified between WBGT and core temperature of agriculture workers |  |
| Jayasekara et al. 2019 | Agricultural workers (257)  No CKD or diabetes (DM) group: 216  CKD or DM group: 41  Mean age: 53.73 ± 11.20 | Mean age: 46.59;13.02  Mean age: 53.73;11.20 | *Workers reporting CKD or DM had:*   - Higher heat stress and dehydration symptom index (10.78 vs. 8.03, p < .01) - Higher (Urine albumin-creatinine ratio) ACR > 30 (85.4% vs. 69.4%, p < .05)   *Among workers with no CKD or DM group:*   - ACR > 30 (high-prevalence CKD region 72.2% vs low prevalence region 55.6%, p < .05) - Higher index (8.4 vs. 6.1, p < .001) | *Association with lower index:*   - Higher income (2.1 fewer points on the index, p < .05) | *Association with higher index:*   - Female status (3.1 additional points on the index, p=.001) | - Age - Average water intake (l) - BMI - ACR > 30 |  |
| Kearney et al. 2016 | 158 farmworkers | 154 males  1 female  Age range: > 18 | - 72% of farmworkers experienced at least one HRI symptom - 27% of farmworkers had three or more HRI symptoms   *HRI symptoms reported:*   - 43.6% headache - 37.6% heavy sweating - 35.7% muscle cramps or spasms - 17.9% weakness or fatigue - 13.5% dizziness or light headedness - 12.1% rash/bumps - 8.5% nausea or vomiting - 4.3% fainting - 1.4% confusion - 5.0% other symptoms or illness during a hot day at work | *Associated with reduced HRI (≥3 Symptoms):*   - Wearing long-sleeved shirts (Prevalence ratio (PR) 0.89, 95% CI: 0.32, 2.51), - Limiting time in sun (PR 0.97 95% CI: 0.46, 2.07) - Wearing sunglasses (PR 0.84, 95% CI:0.33, 2.16) - Wearing long pants (PR 0.35, 95% CI: 0.02, 7.67) | *Associated with increased HRI (≥3 Symptoms):*   - Wearing sunscreen (PR 3.08, 95% CI: 0.87, 10.92) - Wearing a hat (PR 1.39, 95% CI:0.16, 11.87) - Wearing a shirt with collar (PR 1.30, 95% CI: 0.46, 3.62) - Protection over face (PR 1.24, 95% CI: 0.59, 2.60) | - Gloves | *Sun safety behavior used often or always:*   - 98.1% wore long pants - 92.9% wore head protection (baseball hat, cap, or visor) - 27.5% wore a wide-brim hat - 85.8% wore a long-sleeved shirt or blouse - 79.4% wore a shirt with a collar - 56.3% wore gloves - 15.6% wore any protective gear over face - 11.2% wore sunglasses - 9.1% wore sunscreen |
| Kiatkitroj et al. 2021 | Sugarcane farmers (200) | Mean age: 54.9  Male: 79  Female: 121 | - 48% had HRI symptoms - 30.4% of men reported HRI compared to 59.5% of females   *HRI symptoms*   - 81.5% heavy sweating - 71.5% weakness/fatigue - 60% dizziness - 55.5% muscle cramps - 52.0% headache - 51.5% vertigo - 40.5% rash on skin - 36.0% irritability - 18.5% nausea - 16.5% dry and cracking skin - 15% vomiting - 10.5% swelling hands and feet - 9.0% blisters on skin - 4.0% fainting - 7.5 other abnormal symptom | *Association with HRI symptoms*   - Fluid intake 3.1–5.0 L/day (OR: 0.23; 95% CI: 0.09–0.56)   *Association with HRI symptoms*   - Fluid intake of 3.1–5.0 L per day (OR: 0.21, 95% CI: 0.07–0.60) | *Association with HRI symptoms (Univariate analysis):*   - Female sex (OR = 3.37, 95% CI: 1.85–6.141) - Duration of heat exposure ≥ 5 hours/day (OR = 1.88, 95% CI: 1.02–3.44) - Afternoon napping > 60 minutes (OR:2.55, 95% CI: 1.08–5.98) - Wear two long-sleeved shirts (OR: 7.54, 95% CI: 2.92–19.44) - Wear short-sleeved and a long-sleeved shirt (OR: 5.23, 95% CI: 2.23–10.78) - Wear two-layer pants (OR: 2.25, 95% CI: 1.07–4.74)   *Association with HRI symptoms (multivariate analysis):*   - Female sex (OR: 2.37: 95% CI: 1.11–5.04) - Wear two long-sleeved shirts (OR: 5.24, 95% CI: 1.69–16.24) compared to one long-sleeved shirt - Wear short-sleeved and a long-sleeved shirt (OR: 3.61, 95% CI: 1.52–8.53) compared to one long-sleeved shirt | *Univariate analysis:*   - Age - Work experience - Fat - Total Body water - Alcohol consumption - Caffeine intake - Smoking - Hours of sleeping - Duration of heat exposure (<5 hours/day) - Afternoon napping (<60 minutes) - Fluid intake (1-3 L/day) - Wear one-layer polyester or jean pants - Full face mask - Head protection   *Multivariate analysis:*   - Duration of heat exposure - Afternoon napping - Fluid intake (1-3 L/day) - Wear one-layer or two layer pants | - 36.5% consume 1 to 3 L of fluid per day - 50% consume 3.1 to 5 L of fluid per day   13.5% consume more than 5 L of fluid per day |
| Kupferman et al. 2018 | Sugarcane workers (326)  Cane cutters: 153  Seeders/ seed cutters: 59  Weeders: 35  Pesticide applicators:25  Irrigators:54  First follow-up: 29 | Male: 326  Age 18-60 | - 34 participants (10.4%) had AKI - 19.0% of cane cutters had AKI compared with 2.9% performing other job tasks (P < 0.001) - Median serum creatinine (SCr) of the 34 AKI workers was 1.64 vs non-AKI workers 0.88 mg/dL - AKI workers had an increase in median SCr to 1.25 and 1.27 after 6 and 12-month follow-up respectively - 10 participants (34.5%) with AKI developed eGFR < 60 and 11 (37.9%) had >30% decrease in eGFR at 12-month follow-up |  | *Association with higher serum creatinine levels:*   - Job as cane-cutter vs other (e^β^ 1.20, 95% CI 1.13-1.27)   Addition of pre-harvest Scr level to the model:   - Job as cane-cutter vs other 1.16 e^β^, 95% 1.10-1.22) |  |  |
| Kwon et al. 2015 | Farmers (120) | 49 males  71 females  Age: 61;11.6 |  |  |  |  | *Farmworkers wear less clothing with higher WBGT:*   - Clothing insulation (χ2 = 390.923, df = 344, p = 0.041) and had a correlation with WBGT (τ = −.191) - Number of layers for Upper clothing (χ2 =27.880, df = 16, p = 0.033) and had a correlation (τ = −.257) - Footwear (χ2 = 67.726, df = 40, p =0.004) and had correlation (τ = −.123) |
| Lam et al. 2013 | Latino farmworkers (35) | 21 males  14 females  Age range: > 18 |  |  |  |  | - In addition to water participants also reported drinking energy drinks to increase alertness and productivity - Workers identified removal of clothing layers as an HRI preventive measure - Female participants also reported wearing darker clothing and layered clothes to sweat to lose weight |
| Laws et al. 2016 | Sugarcane workers (284) | 251 males  33 females  Age range: 18-63 | - eGFR was 113 mL/min/1.73 m2 - <5% of workers had albuminuria - Men had NGAL and IL-18 levels that were about one-third those of women at both the pre- and late harvest - Increase in NGAL levels among cane cutters (preharvest 7.6 ug/g, late harvest 19.3) - Decrease in NAG levels in factory workers (preharvest 2.23, late harvest 0.60) | *Consumption of electrolyte packet among cane cutters:*   - 23% decrease in mean NGAL (RM, 0.77; 95% CI, 0.61-0.98) - 16% decrease in mean NAG (RM 0.84 95% CI 0.70-1.01)   *Consumption of electrolyte packet among seed cutters:*   - 31% decrease in mean IL-18 (RM 0.69 95% CI, 0.46-1.04)   33% decrease in mean NAG (RM 0.67 95% CI 0.44-1.02) | *Association with higher NGAL among all workers:*   - Cane cutters (RM, 2.57; 95% CI, 1.54-4.27) - Irrigators (RM, 2.07; 95% CI, 1.24-3.47) - Field workers (RM, 1.49; 95% CI, 1.06-2.09) compared to non-field workers   *Association with higher IL-18 among all workers:*   - Field workers (RM 1.61; 95% CI, 1.12-2.31) compared to non-field workers - Cane cutters (RM, 1.89 95% CI 1.08-3.29) - Seeders (RM, 2.11; 95% CI 1.14-3.92) | - Agrichemical applicators did not have increases in kidney injury biomarkers compared to other field workers - No overall association between self-reported daily intake of water or electrolyte solution and NGAL, IL-18, or NAG |  |
| Laws et al., 2015 | Sugarcane workers (284)  Cane cutters (51)  Seeders (36) Seed cutters (19) Agrochemical applicators (29)  Irrigators (49)  Drivers (41) Factory workers (59) | Male: 251  Female: 33  Age: 18-63 | *Cross harvest eGFR* (*ml/min/1.73):*   - Decline in cross harvest eGFR among field workers (-6.9 ml/min) compared to non-field workers - Decline in cross harvest eGFR among seed cutters (-8.6), irrigators (-7.4), cane cutters (-5.0) compared to factory workers - Fewer than 5% of workers had albumin-to-creatinine ratio (ACR) >30 mg/g | *Late harvest eGFR:*   - Consumption of electrolyte packet among cane cutters (6.1 ml/min/1.73 m2; 95% CI: 20.06, 12.2)   *Cross harvest eGFR:*   - Consumption of electrolyte packet among cane cutters (7.0 ml/min/1.73 m2; 95% CI: 1.9, 12.1) | *Decline in pre-harvest eGFR:*   - Number of years employed at the company (0.3 ml/min/1.73 m2 95% CI: 20.6, 20.04) |  |  |
| López-Gálvez et al. 2021 | Farmworkers (101)  Organic fields: 50  Conventional fields: 51  Reference group (50) | Age range: 18-59  Male: 101  Age range: 18-59  Male: 50 | - One farmworker developed kidney disease, two suffered a kidney injury, and 14 were at risk of a kidney injury - Decline in eGFR from pre-harvest (125 ± 13.0) to late harvest (106 ± 17.5) among farmworkers - Significant decline in eGFR among conventional field (101.2 ± 19.4) compared to organic field farmworkers (110.9 ± 13.6) |  | *Association with decline in eGFR:*  Model 1   - Late harvest (β: - 16.98; 95%CI: −20.22, −13.69)   Model 2   - PSI (β: -0.16; 95% CI: −0.15, −0.06) - Mildly Dehydrated (β: -9.68; 95% CI: −13.31, −3.26) - Dehydrated (β: -13.62; 95% CI: −16.56, −7.71)   Model 3   - Organic field (β: -19.02; 95% CI: −27.29, −10.82 - Conventional field (β: -29.17; 95% CI: −37.26, −21.17)   Model 4   - Organic field (β: -0.23; 95% CI: −0.41, −0.03) for every 1% increase in PSI - Conventional field (β: -0.37; 95% CI: −0.57, −0.16) for every 1% increase in PSI - Mild Dehydration (β: -8.51; 95% CI: −13.98, −3.03) - Dehydration (β: -11.99; 95% CI: −16.88, −7.10) | *Model 2:*   - Daily Water Intake (>4 L)   *Model 3:*   - Insecticide application   *Model 4:*   - Daily Water Intake (>4 L) - Insecticide application |  |
| Lumingu et al. 2009 | Young farmworkers (18) | Age range: 12-35 | - All the final sublingual temperatures were under 38°C - Only 4 cases showed sweating that reached 5% of body weight - Mean heart rate was below threshold when WBGT was <28 °C and exceeded threshold when WBGT was >43 °C |  |  |  |  |
| Lundgren et al. 2014 | Agricultural workers (4) | 1 Male  3 Female  Age range: not specified | - Predicted time to reach a core temperature of 38°C was 240 minute in agriculture for females - Average metabolic rate was 190 while doing the following work tasks: Preparation of land for cultivation, sowing, watering, weeding, pest control, fertilization, crop maintenance and harvesting, bending, walking speed 2.45.5 km/h |  |  |  |  |
| Luque et al. 2019 | Hispanic farmworkers (29) | 15 males  14 females  Age range: >19 |  |  | - Farmworkers working under contractual arrangements were less likely to take sufficient breaks and worked for long hours - Focus groups agreed that inexperienced or new workers in the fields are at higher risk for HRIs since they had not had time to acclimatize to the weather conditions |  | - Used a variety of PPE including protective clothing, such as caps, gloves, long sleeve shirts and pants - Brought their own water since they did not like the odor or taste of the tap water at the worksite - Drank electrolyte solutions or Gatorade - Rested in the shade - Used wet cool cloths |
| Luque et al. 2020 | Hispanic farmworkers (101) | 61 males  40 females  Age range: 19-66 | - 19% of participants reported HRI symptoms   *HRI symptoms reported:*   - 2% dizziness - 5% skin rash - 1% muscle cramps - 4% light-headedness - 14% headache - 12% heavy sweating - 3% extreme weakness - 3% nausea - 1% dry skin |  |  |  | *Type of beverages ingested:*   - 89% water - 64% Gatorade - 19% energy drinks - 27% fruit juice - 12% coffee or tea - 26% Soda - 2% Beer - 58% began with a few hours of work before starting to work a full day - 77% used shade under trees - 20% used shade structures - 13% used fans - 10% used rest stations - 11% wore wet hats or bandannas - 66% drank more water - 21% changed work hours - 2% used a vehicle with air conditioning - 23% changed work activities - 23% took rest breaks in the shade   *Clothing always or usually worn:*   - 23% light-colored short-sleeved shirt - 3% dark-colored short-sleeved shirt - 79% light-colored long-sleeved shirt - 5% dark-colored long-sleeved shirt - 5% shorts - 83% pants - 24% jacket   *Head protection always or usually worn:*   - 85% baseball cap - 22% wide-brimmed hat - 3% other hat - 60% bandanna - 8% hood from sweatshirt |
| Mac et al. 2016 | Fernery workers (40) | 13 males  30 females  Age range: 18-54 | - Body core temperature exceeded 38.0°C on 57% of the 86 workdays examined - 30 out of 40 participants had core body temperature that exceeded 38.0°C at least one workday |  | *Associated with an increase in the odds of the body temperature reaching or exceeding 38.0°C:*   - Females (OR 5.38, 95% CI 1.58,18.30) compared to males - Workday energy expenditure (OR 1.12, 95% CI 1.03, 1.21) | - Age - BMI - Body mass - Body surface area - Years working in ferneries - Average WBGT - Workday duration |  |
| Mac et al. 2019 | Fernery workers (40) | 13 males  30 females  Age range: 18-54 | - Body core temperature exceeded 38.0°C on 57% of the 86 workdays examined - 30 out of 40 participants had core body temperature that exceeded 38.0°C at least one workday |  | *Associated with an increase in the odds of the body temperature reaching or exceeding 38.0°C (bivariate models):*   - Total workday energy expenditure (kcal) (OR: 1.08; 95% CI 1.01, 1.15)   *Associated with an increase in the odds of the body temperature reaching or exceeding 38.0°C (multivariate models):*   - Females (OR 5.38, 95% CI 1.58,18.30) compared to males - Workday energy expenditure (OR 1.12, 95% CI 1.03, 1.21) | *Bivariate model:*   - Female sex - Age - BMI - Body mass - Body surface area - Years working in ferneries - Average WBGT - Workday duration   *Multivariate model:*   - Age - BMI - Body mass - Body surface area - Years working in ferneries - Average WBGT   Workday duration |  |
| Miller, 1982 | Tractor driver (1) | Gender: Male  Age range: not specified | - Resting pulse increased from 72 per minute to 140 per minute - Skin temperature increased to 38.4 °C - Sweat loss was 2.54 kg |  |  |  |  |
| Mirabelli et al. (2010) | Latino farmworkers (281)  H-2A workers:177  Non-H2-A workers: 104 | Gender: Not specified  Age range: 18-65 | - 40 % of participants working in extreme heat reported at least one HRI symptom - 31% of H-2A workers reported HRI compared to 56% of non-H2A workers | *Associated with lower HRI:*   - Change work hours and activities among H-2A workers (PR = 0.44, 95% CI=0.22, 0.89) | *Associated with higher HRI:*   - Change work hours and activities among non–H-2A workers (PR=1.11, 95% CI=0.79, 1.55) | Among both H-2A and non-H-2A workers:   - Drink more water - Rest in shaded areas - Go to air-conditioned   places during or after work | - Change work hours (35% H2-A workers vs 40% non-H2-A workers) - Change work activities (30% vs 41%) - Change hours and activities (29% vs 39%) - Drink more water (97% vs 99%) - Take rest breaks in shaded areas (73% vs 93%) - Go to air-conditioned places during breaks or after work (<1% vs 5%)   Change hours or activities (35% vs 43%) |
| Mitchell et al. 2017 | Farmworkers (588) | 389 males  198 females  Age range: 18 – 82 | - 8.3% experienced a body temperature of ≥ 38.5°C - 11.8% experienced dehydration (loss of more than 1.5% of body weight) - 18% exceeded threshold for heart rate for 5 minutes or more |  |  |  |  |
| Mix et al. 2018 | Agricultural workers (192) | 76 males  116 females  Mean age: 38.0;8.2 | - 33% of participants had AKI on at least one workday - 53% of workers were dehydrated (USG 1.020) pre-shift and 81% post-shift - 3% had USG > 1.030) preshift, indicating a clinically dehydrated state which increased to 13%, post-shift - 31% of participants had at least 1 day with a creatinine level above sex specific limits or an increase of 0.3 mg/dL during the day |  | *Association with higher AKI:*   - Heat index (OR=1.47, 95% CI: 1.14 to 1.90) | - Age - Gender - Nationality - Education - BMI - Hypertension - Blood pressure - Diabetes - Work type - Years worked in agriculture - Hours worked per day - Drinks more sports drinks at work - Drinks more energy drinks at work - Drinks more juice at work - Drinks more soda at work | *Type of beverages consumed:*   - 98% water - 69% sports drinks - 50% soda - 39% juice - 16% energy drinks - 9% coffee - 2% alcohol |
| Mohammadian et al. 2020 | Date harvesting workers (59) | Mean Age: 39.3  Male: 36  Female: 23 | - Average systolic blood pressure was 117.81 - Average diastolic blood pressure was 76.01 - Average physiological strain index (PSI) was 2.28 - Average perceptual strain index (PeSI) was 6.61 |  | *Association with PSI:*   - WBGT (correlation coefficient 0.43) - ESI (0.59) - DI (0.60) | - No significant relationship between environmental indices and PeSI |  |
| Moyce et al. 2016 | Agricultural  workers (295) | Male: 190  Female:105 | - 35 participants (11.8%) after a single work shift had cumulative incidence of AKI (stage 1 or stage 2) |  | *Association with AKI:*   - Piece-rate work (AOR 4.52, 95% CI 1.61 to 12.70) | - Sex - Age - BMI - Diabetes status - Blood pressure - Level of education - Years in agricultural work - Farm task |  |
| Moyce et al. 2017 | Agricultural workers (283) | 182 males  101 females  Age range: > 18 | - 12.4% of participants had AKI - AKI was present in 12.1% among men and 12.9% among females - 11.7% experienced heat strain among those who had AKI - 64.8% of men and 52.5% of women lost <1.5% body mass | *Association with lower AKI:*   - Obesity (OR 0.29, 95% CI 0.10 to 0.82) - Overweight men (OR 0.29, 95% CI 0.08 to 0.97) - Obese men (OR 0.25, 95% CI 0.07 to 0.94) | *Association with higher AKI:*   - Heat strain among men (OR 1.31, 95% CI 1.01 to 1.70) - Piece rate work (OR 4.24, 95% CI 1.56 to 11.52) - Piece rate work among females (OR 102.81, 95% CI 7.32 to 1443.20) - Years in agricultural work among females (OR 1.12, 95% CI 1.01 to 1.24) | - Volume depletion (% body mass lost) - Age - Gender - BMI - Diabetes - Blood pressure - History of kidney disease - Farm task |  |
| Moyce et al. 2019 | Agricultural workers (471) | 298 males  173 females  Age range: >18 | - 14.9% of participants had AKI (indicated by increase in serum creatinine of ≥0.3 mg/dL or ≥1.5 times the preshift creatinine) after a single day of agricultural work - 36% had elevations of core body temperature ≥1°C |  | *Association with higher AKI:*   - Workload (1.92; 95% CI 1.05‐3.51) - Piece‐rate work (3.02; 95% CI, 1.44‐6.34) | - Age - Gender - Diabetes - BMI - Blood pressure - History of kidney disease - Max daily WBGT - Years in agricultural work - Farm task |  |
| Moyce et al. 2020 | Agricultural workers  (445) | 283 males  162 females  Age range: >18 | *Compared to females, more males:*   - Experienced AKI (15.9% vs 12.9%) - Had a change in core body temperature that was greater than or equal to 1°C (131 vs 39) - Lost at least 1.5% of their body mass (dehydration indicator) (43 vs 4) |  | *Association with higher odds of AKI:*   - Total volume consumed (AOR 1.47, 95% CI= 1,.9–1.99) - Maximum workload (1.01, 95% CI 1.01–1.02). - Picking (AOR 2.51, 95% CI 1.39–4.54) - Male with diabetes (AOR 6.76, 95% CI 1.49–30.77) - Male picking (4.12, 95% CI= 1.87–9.08) | - Age - BMI - Diabetes - Blood pressure - Payment method - Years in agricultural work - Maximum WBGT - Percentage body mass lost - Sugary drinks | - Men drink 2.9L of water compared to 1.9L for women |
| Mutic et al. 2018 | Farmworkers (198) | 78 males  120 females  Age range: 19-54 | - 84% of participants reported experiencing at least one symptom - 40% reported three or more symptoms   *HRI symptoms reported:*   - 66% heavy sweating - 58% headache - 32% dizziness - 30% muscle cramps - 24% nausea/vomiting - 10% fainting - 9% confusion   *Symptoms fell into three latent classes:*   - Mild (heavy sweating; class probability = 54%) - Moderate (heavy sweating, headache, nausea-vomiting and dizziness; 24%) - Severe (excessive sweating, headache, dizziness, nausea-vomiting, sudden muscle cramps and fainting; 22%) |  | *Association with higher odds of HRI:*   - Female (OR = 2.86, 95% CI 1.18–6.89) | - Age - Nationality - BMI - Reported hypertension or diabetes - Education - Alcohol - Smoking - Work type - Days worked per week - Hours worked per day |  |
| Nanayakkara et al. 2020 | Paddy farmers (25) | Male: 25  Age: 23-64 | - 12% and 4% of farmers in the morning had dehydration in non-farming and farming seasons respectively according to urine osmolarity - 24% and 40% of farmers in the evening showed dehydration in the non-farming and farming seasons respectively urine osmolarity - 88% and 72% of farmers in the morning had dehydration in non-farming and farming seasons respectively according to plasma osmolarity - 2% and 40% of farmers in the evening had dehydration in non-farming and farming seasons respectively according to plasma osmolarity |  |  |  |  |
| Nayha et al. 2017 | Working population (4007)  Agriculture: 136 (3.4%) | 1860 males  2147 females  Age range: 25-74 |  |  | *Association with higher odds of cardiorespiratory symptoms:*   - Working in agriculture (OR 2.27; 1.14–4.46) compared with working in industry |  |  |
| Pogacar et al. 2017 | Farmers (230) | 143 males  87 females  Age not specified | *HRI symptoms reported:*   - Headache (women 64% vs men 46% %) - Exhaustion (69% vs 56%) - Nausea or vomiting (19% vs 8 %) - Fainting (11% vs 6%) - Prickly heat (8 % vs 13 %) - Muscle cramps (2 % vs 6 %) - Heat cramps (0 % vs 0.7 %) |  |  |  | - 79% drank more water - 44% change to lighter/less clothing - 54% took breaks in cooler areas - 34% took more breaks - 73% adjusted work schedule |
| Pundee et al. 2021 | Sugarcane farmers (98)  Sugarcane cutters (150) | Male: 37  Female: 61  Male: 66  Female: 84 | - % Cross-shift change in urine creatinine (92.7% vs 85.65%) - Post-shift NGAL (8.93 vs 11.35) - %Cross-shift change in NGAL (182% vs 112%) |  | *Association with higher post-shift NGAL (univariate analysis)*   - Sugarcane cutter (OR: 0.42; 95% CI: 0.22–0.71) - Working hour (4.6–8.45 h) (OR: 0.41; 95% CI: 0.19–0.87)   *Association with higher post-shift ACR (multivariate analysis)*  Water intake ≤ recommendation (OR: 5.33; 95% CI: 1.04, 27.33) | *Association with higher post-shift ACR*   - Sugarcane cutter (reference: Sugarcane farmers) - Working hours - Water intake > recommendation   *Association with higher post-shift NGAL*   - Sugarcane cutter (reference: Sugarcane farmers) - Working hours - Water intake > recommendation - Water intake ≤ recommendation | - 36% of males consume the recommended amount of water (>3.7L) - 74% of females consume the recommended amount of water (>2.7L) |
| Quandt et al. 2008 | Migrant Latino farmworkers (304) | 300 males  4 females  Age range: 18-70 | - Effects on skin related quality of life (QOL) measured using the Dermatology Life Quality Index (DLQI) were reported in 38.7% of observation |  | *Engaging in specific work tasks was associated with elevated DLQI*   - Planting (OR: 4.16, CI 2.53–6.84) - Cultivating (OR: 2.39, CI 1.17–4.86) - Tobacco topping (OR: CI 2.44, 1.44-4.13) - Harvesting (OR: 2.20, CI 1.46–3.31)   *Working in higher temperatures was associated with elevated DLQI*   - Working in temperatures > 23.9°C and ≤26.7°C (OR: 2.29, CI 1.21–4.36) - Working in temperatures >26.7°C (OR: 3.33, CI 1.37–8.12) | - Self-rated health - Barning tobacco |  |
| Raines et al. 2014 | People with occupational history in agriculture (151 out of a total sample 424) | 166 males  258 females  Age range: 15–69 | - 77% of agricultural workers had reduced eGFR |  | *Association with reduced GFR among current or former agricultural workers (univariate analysis):*   - Male sex (p <0.001) - Lifetime days cutting or harvesting crops (p=0.004) - Lifetime days cutting or harvesting sugarcane (p=0.005) - Lifetime hours cutting or harvesting sugarcane in the dry season (p=0.001)   *Association with reduced GFR among agricultural workers (multivariate analysis):*   - Any lifetime history cutting sugarcane during the dry season (OR 4.07, 95% CI 1.32–12.58) - Pesticide inhalation (3.14, 95% CI 1.12–8.78) - Sugarcane chewing (OR 3.12, 95% CI 1.21–8.04) - Daily bolis at work (OR 1.48, 95% CI 1.02–2.14) | *Univariate analysis:*   - Hypertension - Age - Diabetes - Nephrotoxic medications - Smoking - Fructose intake - Sugary beverage intake - Daily bolis at work - Water consumption - Alcohol consumption - Lifetime days seeding - Lifetime days watering - Lifetime days mixing pesticides - Lifetime days applying pesticides - Lifetime days working in fields with pesticide use - Inhaled pesticides - Cane chewing - Worked near burning sugarcane   *Multivariate analysis:*   - Age - Male sex - Systolic blood pressure - Alcohol consumption - >365 lifetime days harvesting any crop |  |
| Rajewski et al. 2008 | Agricultural worker (1) | 1 male  Age: 56 | - Male agricultural worker working in the field for about 8 hrs without breaks, fluid intake and no PPE died of heat stroke.   *Examination of patient revealed:*   - Red skin - Body temperature: 41.2 ° C - Heart rate: 160 / min - Blood pressure: 130/80 mm Hg |  |  |  |  |
| Raju et al. 2014 | CKD Patients (198)  Renal insufficiency (656) | Mean age: 46.64 (11.63) | - 24% of CKD patients were agricultural workers - Reduced GFR with increasing age   *CKD patients compared to controls:*   - Significant decrease (p<0.001) in creatinine clearance - Increase in blood urea (98.77 vs 28.55 mg/dl) - Increase in serum creatinine (4.6 vs 0.9 mg/dl) |  |  |  |  |
| Ricco et al. 2017 | Pesticide applicators (131) | 107 males  24 females  Age range: > 18 | - 41.2% of participants reported 3 or more symptoms - 93.1% had at least one symptom   *HRI symptoms reported:*   - 79.6% profuse sweating - 29.0% asthenia - 8.4% confusion, feeling disoriented - 8.4% nausea and/or vomiting - 13.8% high heart rate - 37.4% headache - 15.3% dizziness - 23.7% fainting, rapid breathing - 8.4% blurred vision - 7.7% sudden loss of postural tone without even temporary loss of consciousness - 3.1% sudden loss of postural tone with even temporary loss of consciousness |  | *Associated with higher HRI (univariate analysis):*   - Female sex (OR 3.632, 95%CI 1.392–9.135) - Being a professional farmer (OR 2.438, 95%CI 1.168–5.058) - Drink at least one glass of water every working shift (OR 0.059, 95%CI 0.016–0.211 - Drink at least five glasses of water every working shift (OR 2.753, 95%CI 1.327–5.709) - Alcohol consumption (OR 3.339, 95%CI 1.513–7.367) - Take rest breaks in shady, not air-conditioned areas (OR 4.174, 95%CI 1.473–11.828) - Perform pesticide application (OR 2.705, 95%CI 1.296–5.646) - Manual hoeing/weeding (OR 2.975, 95%CI 1.185–42.035)   *Associated with higher HRI (multivariate analysis):*   - Manual hoeing/ weeding (OR:8.847 95% CI 1.882–41.579) - Pesticide application (OR 8.847, 95% CI 1.882–41.579) - Rests in shady, not air-conditioned areas (OR:5.491 95% CI 1.372–21.971) | *Univariate analysis:*   - Age - Seniority - Migration background - Education - ≥ 3 days a week - Seeding - Harvesting/Picking - Machine operation - Irrigation - Hoeing/weeding, mechanized - Pruning - Previous training about high temperatures - Drink more water - Take rest breaks in cooler, air-conditioned areas - Increased number and/or frequency of rest breaks - Anticipate/Delay hours of work activities   *Multivariate analysis:*   - Age - Female sex - Seniority - Migration background - Education - Being a professional farmer - ≥ 3 days a week - Seeding - Harvesting/Picking - Machine operation - Irrigation - Hoeing/weeding, mechanized - Pruning - Previous training about high temperatures - Drink at least one glass of water every working shift - Drink at least five glasses of water every working shift - Alcohol consumption - Drink more water - Take rest breaks in cooler, air-conditioned areas - Increased number and/or frequency of rest breaks - Anticipate/Delay hours of work activities | - 38.9% received HRI training during the previous 5 years - 90.8% drank water - 78.6% took rest breaks in shady, not air conditioned areas - 23.7% took rest breaks in cooler, air-conditioner areas - 66.4% increased number and/or frequency of rest breaks - 77.1% anticipated/delayed hours of work activities   *Sun protection behavior:*   - 72.5% used head protection - 61.1% used sunscreen - 93.1% used long sleeves shirts - 89.3% used specific clothes - 84.7% used sunglasses |
| Sadiq et al. 2019 | Maize farmers (396) | 251 males  145 females  Age range: 15-60 | *HRI symptoms reported everyday:*   - 93.2% heavy sweating - 48.5% tiredness - 34.1% dizziness - 40.4% headache - 29.8% heat rash/pricking - 25.3% rapid pulse - 33.8% nausea/ vomiting - 19.7% elevated body temperature - 9.8% muscle cramp - 11.1% fainting |  |  |  |  |
| Sahu et al. 2013 | Rice harvesters (124) | 124 males  Age range:18-45 | *Physiological symptoms:*   - Heart rate recovery is fast in low temperature and slow in high temperature after work period indicating cardiovascular strain - Higher peak heart rate, sum of recovery heart beats and cardiac cost variables with increasing temperature   *HRI symptoms reported:*   - 37% discomfort - 50% heat exhaustion - 72% pain in different body parts |  |  |  |  |
| Sandsund et al.2021 | Fish farmers (14) | Age range: 16-64  Male:13  Female:1 | - Minimum Tc was 37.1 ± 0.2 °C - Maximum Tc was 38.1 ± 0.2 °C - Skin temperatures ranged from 18 °C (front thigh) to 35 °C (chest) |  |  |  |  |
| Sen & Nag 2019 | Farmworkers (1144) | Males:632  Females:512  Age range: 30-50 | - 1/3 complained of moderate to high intensity of back pain, muscle pain and heavy sweating. - Females had more marked heat-related symptoms compared to males - 11% and 6% of female and male farmers, respectively had symptoms of tachycardia, dizziness, headache, and blurring of vision |  |  |  |  |
| Silpasuwan et al. 2020 | Salt farmworkers (120) | Mean Age: 49.5  Female: 73  Male:47 | *HRI symptoms*   - Heat stroke (everyday 0, someday 6.6, never 93.4) - Heat cramps 0.9, 15.1, 84.0) - Heat rash (0.9, 20.8, 77.4) - Heat exhaustion (0, 49.1, 50.9) |  | *Association with healthy skin*   - Heat Exposure (β: 0.35, p=0.01) |  |  |
| Smith et al. 2020 | Migrant farmworkers (60) | 49 males  11 females  Age range: 18-51 | - 68% of participants had one or more HRI symptoms - 12% had 3 or more symptoms   *HRI symptoms reported:*   - 50% heavy sweating - 25% cramps - 22% headache - 10% dizziness - 3% nausea |  |  |  | - 43.76% drank water - Mean liquid consumption was 72.95 oz per day, which is much less than the recommended 32 oz per hour |
| Sorensen et al. 2019 | Sugarcane workers  (105) | Age range: >18 | - Average decline in cross-shift eGFR was 21.8% - 31% to 51% of workers had post-shift serum osmolality less than 280mmol/kg - Average albumin to creatinine ratio (ACR) ranged from 9 to 22mg/mg pre-shift to 18 to 30mg/mg post-shift and only increased significantly across the work shift in March |  | *Association with reduced cross-shift GFR (univariate analysis):*   - Age (estimate -0.3, p<0.01) - Harvests worked (-0.5, p<0.01) - HbA1c (-7.8, p=0.02) - BUN (-0.9, p <0.01) - Serum osmolality (-0.3, p= 0.01) - Pre-shift urine specific gravity (-2.6, p<0.01) - Post-shift urine specific gravity (-3.3, p<0.01)   *Association with reduced cross-shift GFR among post-shift biomarkers:*   - Average WBGT (-1.6231, p=0.03) - Increasing age (-0.4177, p<0.01)   *Association with reduced cross-shift GFR among pre-shift biomarkers:*   - Diabetes indicator HbA1c (-7.35, p=0.04) - Increasing Age (-0.26, p= 0.04) | *Univariate analysis:*   - Highland - BMI - Hypertension - NSAID - Smoking - Preshift GFR - Average WBGT - Maximum WBGT - Cane harvested on study day/previous day - Urine biomarkers (   *Multivariate analysis among pre-shift biomarkers:*   - Hypertension - Pre-shift GFR - Pre-shift specific gravity   *Multivariate analysis among post-shift biomarkers:*   - HbA1c - Osmolality |  |
| Spector et al. 2015 | Crop workers (97) | 51 male  46 female  Age range: > 18 | - 1/3 participants reported HRI symptoms which include dizziness/light-headedness and heavy sweating | *Associated with lower HRI:*   - - Age (OR = 0.92; 95% CI=0.87–0.98) | *Associated with higher HRI:*   - Piece rate (OR = 6.20; 95% CI = 1.11–34.54) - Walking for more than 3 minutes to get to the toilet (OR = 4.86; 95% CI = 1.18–20.06) | - Male - BMI - Diabetes mellitus and/or antihypertensive medication use - Good/fair general health - No HRI Training - No light-colored shirt - Drank caffeine - Drank less than every 30 minutes - Heat index - No Extra breaks - Hard/very hard work | - 57% drank water every 30 minutes in the past week - More than 75% wore a light-colored shirt |
| Spector et al. 2018 | Harvesters (46) | 39 males  7 females  Mean age: 39.1;14.1 | - Mean pre-shift urine specific gravity was 1.025 which is considered minimal dehydration - 24% exhibited excessive sleepiness   *Significant decrease across shift*   - PVT mean reaction time (t [43] = 3.4, p=0.002) - Number of lapses (t[43]= 2.3, p=0.029) - Mean path length for both eyes open (t[45]= 3.8, p < 0.001) and eyes closed (t[45]= 3.1, p= 0.004) |  |  | - Heat exposure was not associated with impaired vigilance or balance |  |
| Stoecklin-Maroi et al. 2013 | Farmworkers (467) | 263 males  211 females  Age range: 18-55 |  |  |  |  | - 91% received HRI training (87.5% male vs 96.7% female, p=0.0003) - 29.7% of workers bring their own water to work (44.9% vs 10.5%) - Workers drank 10.7 times/ day employer provided beverages (11.1 vs 10.3) |
| Stoklosa et al. 2020 | Migrant Agricultural Worker (1) | Male  Age: 25 | - Elevated creatinine level (3.9 mg/dL) - Serum potassium level of 5.2 mmol/L - Mildly elevated total creatine kinase (219 U/L) - The diagnoses were heat exhaustion, severe dehydration, and renal insufficiency   *Symptoms included:*   - Lightheadedness - Blurred vision - Fasciculation - Nausea - Vomiting - Abdominal cramping |  |  |  |  |
| Trevisan et al. 2019 | Sugarcane workers (32) | 32 males  Mean age: 47.4 | - Rhinitis symptoms were (53.4%) compared to the non-harvesting period (26.7%, p = 0.039) and 6 months after the beginning of harvesting (20%, p = 0.0006) - Significant increase in IL-6 (inflammatory marker) after 3 months of harvesting compared to non-harvesting period (p = 0.012) |  |  |  |  |
| Vega‐Arroyo et al. 2018 | Farmworkers (259) | 168 males  91 females  Age range: >18 | - 45% had a body temperature greater or equal to 38°C - 15% percent of workers were hypohydrated based on loss of 1.5% of body fat |  | *Association with higher CBT:*   - Work rate (β = .006, 95% CI [0.004, 0.009]) - WBGT (β = .03, 95% CI [0.017, 0.05]) | - Gender - Age - Clothing ensemble insulation heat gain - Hydration status (weight loss) - Head gear insulation heat gain |  |
| Venugopal et al. 2021 | Agricultural workers (223) | Mean age: 43.7  Male: 44  Female: 179 | - 9.8% had increase in CBT greater than 1 °C - 4.1% had sweat rate above safe limit for more than 1 l/h - 2.2% had increase in urine specific gravity - 93.7% had HRI symptoms - 73.1% had dehydration - 45.2% had urogenital issues |  |  |  |  |
| Wagoner et al. 2020 | Farmworkers (28) | Age range: >18 | - Mean S.G.s of post-shift March, June and August were 1.25, 1.025, and 1.02, respectively - 40% of June post-shift samples were clinically dehydrated and 57% were mildly dehydrated - The percentage of time workers’ core body temperatures were over the 38 °C was minimal - Body temperatures between 37 °C and 38 °C made up over 80% of the workdays |  |  |  |  |
| Wegman et al. 2018 | Sugarcane workers (80)  Intervention group (Inland): 40  Nonintervention group (Coastland): 40 | Age range: 18-63  Intervention group:  39 males  1 female  Nonintervention group:  28 males  12 females | *Decrease in cross harvest GFR:*   - Intervention group −3.4 ml/min/1.73m2 (95% CI, −5.5 to −1.3) - Nonintervention group -5.3 (95% CI -7.9 to -2.7) - Compared to inland group, more workers had eGFR <60 in the Coastland group at baseline (5 versus 2), and at the end of the harvest (7 versus 2) - Comparing group differences in eGFR (ie, changes from baseline to end of harvest) showed a smaller decrease in the inland group compared with the coastland group that was close to significant; 2.8 percentage points (95% CI -1.1–6.5) |  |  |  |  |
| Wesseling et al. (2016a) | Sugarcane workers (29) | 29 males  Age range: 17–38 | *Cross harvest changes in pre-shift biomarkers:*   - 10% decrease in GFR - 16% increase in creatinine - 40% increase in serum urea N - 4 times increase in NGAL |  |  |  |  |
| Wesseling et al. (2016b) | Three working population groups  (194)  Construction workers: 56  Sugarcane cutters: 86  Small-scale  farmers: 52 | 194 male  Age range: 17–39 | - Reduced eGFR (<80 mL/min/1.73 m2) (sugarcane cutters 16% vs farmers 2%) - Proteinuria >30 mg/dL (14.7% vs 6.1%) - SCr >1.2 mg/dL (17.4% vs 5.8%) - BUN >20 mg/dL (15.1% vs 1.9%) - Leucocyturia (22.1% vs 1.9%) | *Association with improved GFR among sugarcane cutters:*   - Electrolyte solution (β 8.1, 95% CI −1.2 to 17.5, p=0.09) | *Association with reduced GFR among all workers and restricted to sugarcane cutters (univariate analysis):*   - Work as a sugarcane cutter (p=0.007) - High intake of water (p=0.007) - Low intake of sugary beverages (p=0.007) - Increasing age (p=0.002) - Low haemoglobin (p=0.001) - High tobacco consumption (p=0.02)   *Association with reduced GFR among sugarcane workers (univariate analysis):*   - Cumulative time in job   *Association with reduced GFR among all workers:*   - - Age (β −1.3, 95% CI −1.8 to −0.8; p<0.001) - Serum uric acid (mg/dL) (β -10.4, 95% CI −12.2 to −8.5)   *Association with reduced GFR among sugarcane cutters:*   - Age (β −1.9, 95% CI −2.7 to −1.1, p<0.001) - Serum uric acid (mg/dL) (β -11.3, 95% CI -14 to -8.6) | *Association with reduced GFR among all workers and restricted to sugarcane cutters (univariate analysis):*   - Work day (hours), median - Hours cutting cane, median - Total break time (min), median - Breaks ≤2/d, % (# cases) - No shade during breaks, % - High speed perception, % - Production (tons/d), median - Incentives to cut more, % - History of pesticide use - Total fluid intake (L), median - Low total fluid intake - High total fluid intake - Water (L), median - Low water intake - Sugary beverage, median - High sugary drink intake - Intake electrolyte solution - Hypertension - BMI - Alcohol - Nephrotoxic medication   *Association with reduced GFR among all workers and restricted to sugarcane cutters (multivariate analysis):*   - Sugarcane cutter ever - Cumulative time in job - Work day (hours), median - Hours cutting cane, median - Total break time (min), median - Breaks ≤2/d, % (# cases) - No shade during breaks, % - High speed perception, % - Production (tons/d), median (10%; 90%) - Incentives to cut more, % - History of pesticide use - Total fluid intake (L), median - Low total fluid intake - High total fluid intake - Water (L), median - Low water intake - High water intake - Sugary beverage, median - Low sugary drink intake - High sugary drink intake - Hypertension - BMI - Tobacco - Alcohol - Nephrotoxic medication |  |
| Wilmsen et al. 2019 | Latino forestry workers (23) | Mean age: 30 | - One worker had heat illness and didn’t achieve full recovery |  |  |  |  |

**List of included articles**

1. Arcury, T. A., Arnold, T. J., Quandt, S. A., Chen, H., Kearney, G. D., Sandberg, J. C., . . . Daniel, S. S. (2019). Health and Occupational Injury Experienced by Latinx Child Farmworkers in North Carolina, USA. International Journal of Environmental Research & Public Health [Electronic Resource], 17(1), 30.
2. Arcury, T. A., Quandt, S. A., Arnold, T. J., Chen, H., & Daniel, S. S. (2020). Occupational Injuries of Latinx Child Farmworkers in North Carolina: Associations with Work Safety Culture. Journal of Occupational & Environmental Medicine, 05, 05.
3. Arcury, T. A., Summers, P., Talton, J. W., Chen, H., Sandberg, J. C., Johnson, C. R. S., & Quandt, S. A. (2015). Heat illness among North Carolina Latino Farmworkers. Journal of Occupational and Environmental Medicine, 57(12), 1299-1304. doi:10.1097/JOM.0000000000000552
4. Arnold, T. J., Arcury, T. A., Sandberg, J. C., Quandt, S. A., Talton, J. W., Mora, D. C., . . . Daniel, S. S. (2020). Heat-Related Illness Among Latinx Child Farmworkers in North Carolina: A Mixed-Methods Study. New Solutions, 30(2), 111-126.
5. Bethel, J. W., & Harger, R. (2014). Heat-related illness among Oregon farmworkers. International Journal of Environmental Research & Public Health [Electronic Resource], 11(9), 9273-9285.
6. Bethel, J. W., Spector, J. T., & Krenz, J. (2017). Hydration and Cooling Practices Among Farmworkers in Oregon and Washington. Journal of Agromedicine, 22(3), 222-228.
7. Biggs, C., Paterson, M., Maunder, E. Hydration status of South African forestry workers harvesting trees in autumn and winter. Ann Occup Hyg. 2011 Jan;55(1):6-15. doi: 10.1093/annhyg/meq068. Epub 2010 Oct 25. PMID: 20974676.
8. Bodin, T., Garcia-Trabanino, R., Weiss, I., Jarquin, E., Glaser, J., Jakobsson, K., . . . Group, W. E. P. W. (2016). Intervention to reduce heat stress and improve efficiency among sugarcane workers in El Salvador: Phase 1. Occupational & Environmental Medicine, 73(6), 409-416.
9. Budhathoki, N. K., & Zander, K. K. (2019). Socio-Economic Impact of and Adaptation to Extreme Heat and Cold of Farmers in the Food Bowl of Nepal. International Journal of Environmental Research & Public Health [Electronic Resource], 16(9), 06.
10. Butler-Dawson, J., Krisher, L., Asensio, C., Cruz, A., Tenney, L., Weitzenkamp, D....Newman, L. S. (2018). Risk Factors for Declines in Kidney Function in Sugarcane Workers in Guatemala. *J Occup Environ Med, 60*(6), 548-558.
11. Butler-Dawson, J., Krisher, L., Yoder, H., Dally, M., Sorensen, C., Johnson, R. J., . . . Newman, L. S. (2019). Evaluation of heat stress and cumulative incidence of acute kidney injury in sugarcane workers in Guatemala. International Archives of Occupational & Environmental Health, 92(7), 977-990.
12. Centers for Disease, C., & Prevention. (2008). Heat-related deaths among crop workers--United States, 1992--2006. MMWR - Morbidity & Mortality Weekly Report, 57(24), 649-653.
13. Cortez, O. D. (2009). Heat stress assessment among workers in a Nicaraguan sugarcane farm. Global Health Action, 2(1). doi:10.3402/gha.v2i0.2069
14. Crowe, J., de Joode, B. V., & Wesseling, C. (2009). A pilot field evaluation on heat stress in sugarcane workers in Costa Rica: What to do next? Global Health Action, 2, 71-80. doi:10.3402/gha.v2i0.2062
15. Crowe, J., Nilsson, M., Kjellstrom, T., & Wesseling, C. (2015). Heat-related symptoms in sugarcane harvesters. American Journal of Industrial Medicine, 58(5), 541-548.
16. Culp, K., & Tonelli, S. (2019). Heat-Related Illness in Midwestern Hispanic Farmworkers: A Descriptive Analysis of Hydration Status and Reported Symptoms. Workplace Health & Safety, 67(4), 168-178.
17. Das, B., Ghosh, T., & Gangopadhyay, S. (2013a). Child work in agriculture in West Bengal, India: assessment of musculoskeletal disorders and occupational health problems. Journal of Occupational Health, 55(4), 244-258.
18. Das, B., Ghosh, T., & Gangopadhyay, S. (2013b). Prevalence of musculoskeletal disorders and occupational health problems among groundnut farmers of West Bengal, India. Journal of Human Ergology, 42(1-2), 1-12.
19. Ekiti, M. E., Zambo, J. B., Assah, F. K., Agbor, V. N., Kekay, K., & Ashuntantang, G. (2018). Chronic kidney disease in sugarcane workers in Cameroon: a cross-sectional study. BMC Nephrology, 19(1), 10.
20. Fitria, L., Prihartono, N. A., Ramdhan, D. H., Wahyono, T. Y. M., Kongtip, P. & Woskie, S. (2020). Environmental and Occupational Risk Factors Associated with Chronic Kidney Disease of Unknown Etiology in West Javanese Rice Farmers, Indonesia. *Int J Environ Res Public Health, 17*(12).
21. Fleischer, N. L., Tiesman, H. M., Sumitani, J., Mize, T., Amarnath, K. K., Bayakly, A. R., & Murphy, M. W. (2013). Public health impact of heat-related illness among migrant farmworkers. American Journal of Preventive Medicine, 44(3), 199-206.
22. Flocks, J., Mac, V. V. T., Runkle, J., Tovar-Aguilar, J. A., Economos, J., & McCauley, L. A. (2013). Female Farmworkers' Perceptions of Heat-Related Illness and Pregnancy Health. Journal of Agromedicine, 18(4), 350-358. doi:10.1080/1059924x.2013.826607
23. Frimpong, K., Odonkor, S. T., Kuranchie, F. A., & Nunfam, V. F. (2020). Evaluation of heat stress impacts and adaptations: perspectives from smallholder rural farmers in Bawku East of Northern Ghana. Heliyon, 6(4), e03679.
24. Frimpong, K., Oosthuizen, J. d., & Van Etten, E. J. (2013) Experiences Of Heat Stress Vulnerability And Climate Change Among Farmers In Ghana. (AGRIS FAO database)
25. Garcia-Trabanino, R., Jarquin, E., Wesseling, C., Johnson, R. J., Gonzalez-Quiroz, M., Weiss, I., . . . Barregard, L. (2015). Heat stress, dehydration, and kidney function in sugarcane cutters in EI Salvador - A cross-shift study of workers at risk of Mesoamerican nephropathy. Environmental Research, 142, 746-755. doi:10.1016/j.envres.2015.07.007
26. Glaser, J., Hansson, E., Weiss, I., Wesseling, C., Jakobsson, K., Ekstrom, U., . . . Wegman, D. H. (2020). Preventing kidney injury among sugarcane workers: promising evidence from enhanced workplace interventions. Occupational & Environmental Medicine, 77(8), 527-534.
27. Gun, R. T., & Budd, G. M. (1995). Effects of thermal, personal and behavioural factors on the physiological strain, thermal comfort and productivity of Australian shearers in hot weather. Ergonomics, 38(7), 1368-1384.
28. Hansen, A. L., Williams, S., Hanson-easey, S., Varghese, B. M., Bi, P., Heyworth, J., . . . Pisaniello, D. L. (2020). Using a qualitative phenomenological approach to inform the etiology and prevention of occupational heat-related injuries in Australia. International Journal of Environmental Research and Public Health, 17(3). doi:10.3390/ijerph17030846
29. Hansson, E., Glaser, J., Jakobsson, K., Weiss, I., Wesseling, C., Lucas, R. A. I., . . . Wegman, D. H. (2020). Pathophysiological Mechanisms by which Heat Stress Potentially Induces Kidney Inflammation and Chronic Kidney Disease in Sugarcane Workers. Nutrients, 12(6), 02.
30. Hansson, E., Glaser, J., Weiss, I., Ekstrom, U., Apelqvist, J., Hogstedt, C., . . . Wegman, D. H. (2019). Workload and cross-harvest kidney injury in a Nicaraguan sugarcane worker cohort. Occupational & Environmental Medicine, 76(11), 818-826.
31. How, V., Baharudin, N. A. M., Singh, S., Guo, H. R., Dang, Q. T., Chokeli, R., & Yuswir, N. S. (2020). The Different Effects of Climate Extremes on Physiological Health Among Agroecology and Conventional Smallholder Rice Farmers. Environmental Justice, 13(2), 47-54. doi:10.1089/env.2020.0001
32. Jayasekara, K. B., Kulasooriya, P. N., Wijayasiri, K. N., Rajapakse, E. D., Dulshika, D. S., Bandara, P., . . . Albert, S. M. (2019). Relevance of heat stress and dehydration to chronic kidney disease (CKDu) in Sri Lanka. Preventive Medicine Reports, 15, 100928.
33. Kearney, G. D., Hu, H., Xu, X., Hall, M. B., & Balanay, J. A. (2016). Estimating the Prevalence of Heat-Related Symptoms and Sun Safety-Related Behavior among Latino Farmworkers in Eastern North Carolina. Journal of Agromedicine, 21(1), 15-23.
34. Kwon, J., Park, H. S., Kim, S. H., & Lee, K. S. (2015). Impacts of gender, weather, and workplace differences in farm worker's gear. Journal of Physiological Anthropology, 34, 39.
35. Lam, M., Krenz, J., Palmandez, P., Negrete, M., Perla, M., Murphy-Robinson, H., & Spector, J. T. (2013). Identification of barriers to the prevention and treatment of heat-related illness in Latino farmworkers using activity-oriented, participatory rural appraisal focus group methods. BMC Public Health, 13, 1004.
36. Laws, R. L., Brooks, D. R., Amador, J. J., Weiner, D. E., Kaufman, J. S., Ramirez-Rubio, O., . . . McClean, M. D. (2016). Biomarkers of Kidney Injury Among Nicaraguan Sugarcane Workers. American Journal of Kidney Diseases, 67(2), 209-217.
37. Laws, R. L., Brooks, D. R., Amador, J. J., Weiner, D. E., Kaufman, J. S., Ramírez-Rubio, O....McClean, M. D. (2015). Changes in kidney function among Nicaraguan sugarcane workers. *Int J Occup Environ Health, 21*(3), 241-250. doi:10.1179/2049396714y.0000000102
38. Lumingu, H. M. M., & Dessureault, P. (2009). Physiological responses to heat strain: A study on personal monitoring for young workers. Journal of Thermal Biology, 34(6), 299-305. doi:10.1016/j.jtherbio.2009.04.001
39. Lundgren, K., Kuklane, K., & Venugopal, V. (2014). Occupational heat stress and associated productivity loss estimation using the PHS model (ISO 7933): A case study from workplaces in Chennai, India. Global Health Action, 7(1). doi:10.3402/gha.v7.25283
40. Luque, J. S., Becker, A., Bossak, B. H., Grzywacc, J. G., Tovar-Aguilar, J. A., & Guo, Y. (2020). Knowledge and Practices to Avoid Heat-Related Illness among Hispanic Farmworkers along the Florida-Georgia Line. Journal of Agromedicine, 25(2), 190-200. doi:10.1080/1059924x.2019.1670312
41. Luque, J. S., Bossak, B. H., Davila, C. B., & Tovar-Aguilar, J. A. (2019). "I Think the Temperature was 110 Degrees!": Work Safety Discussions Among Hispanic Farmworkers. Journal of Agromedicine, 24(1), 15-25.
42. Mac, V. V. T. (2016). Characterizing Heat-Related Illness in Florida Farmworkers: A Feasibility Study. Characterizing Heat-Related Illness in Florida Farmworkers: A Feasibility Study, 1-1.
43. Mac, V. V. T., Tovar-Aguilar, J. A., Elon, L., Hertzberg, V., Economos, E., & McCauley, L. A. (2019). Elevated Core Temperature in Florida Fernery Workers: Results of a Pilot Study. Workplace Health & Safety, 67(9), 470-480.
44. Miller, C. W. (1982). Heat exhaustion in tractor drivers. Occupational Health, 34(8), 361-365.
45. Mirabelli, M. C., Quandt, S. A., Crain, R., Grzywacz, J. G., Robinson, E. N., Vallejos, Q. M., & Arcury, T. A. (2010). Symptoms of heat illness among Latino farm workers in North Carolina. American Journal of Preventive Medicine, 39(5), 468-471.
46. Mitchell, D. C., Castro, J., Armitage, T. L., Vega-Arroyo, A. J., Moyce, S. C., Tancredi, D. J., . . . Schenker, M. B. (2017). Recruitment, Methods, and Descriptive Results of a Physiologic Assessment of Latino Farmworkers: The California Heat Illness Prevention Study. Journal of Occupational & Environmental Medicine, 59(7), 649-658.
47. Mix, J., Elon, L., Vi Thien Mac, V., Flocks, J., Economos, E., Tovar-Aguilar, A. J., . . . McCauley, L. A. (2018). Hydration Status, Kidney Function, and Kidney Injury in Florida Agricultural Workers. Journal of Occupational & Environmental Medicine, 60(5), e253-e260.
48. Moyce, S., Armitage, T., Mitchell, D., & Schenker, M. (2020). Acute kidney injury and workload in a sample of California agricultural workers. American Journal of Industrial Medicine, 63(3), 258-268.
49. Moyce, S., Joseph, J., Tancredi, D., Mitchell, D. & Schenker, M. (2016). Cumulative Incidence of Acute Kidney Injury in California's Agricultural Workers. *J Occup Environ Med, 58*(4), 391-397.
50. Moyce, S., Mitchell, D., Armitage, T., Tancredi, D., Joseph, J., & Schenker, M. (2017). Heat strain, volume depletion and kidney function in California agricultural workers. Occupational & Environmental Medicine, 74(6), 402-409.
51. Moyce, S., Mitchell, D., Vega, A., & Schenker, M. (2020). Hydration Choices, Sugary Beverages, and Kidney Injury in Agricultural Workers in California. Journal of Nursing Scholarship, 11, 11.
52. Mutic, A. D., Mix, J. M., Elon, L., Mutic, N. J., Economos, J., Flocks, J., . . . McCauley, L. A. (2018). Classification of Heat-Related Illness Symptoms Among Florida Farmworkers. Journal of Nursing Scholarship, 50(1), 74-82.
53. Nanayakkara, I., Dissanayake, R. K. & Nanayakkara, S. (2020). The presence of dehydration in paddy farmers in an area with chronic kidney disease of unknown aetiology. *Nephrology (Carlton, Vic.), 25*(2), 156-162.
54. Nayha, S., Rintamaki, H., Donaldson, G., Hassi, J., Jousilahti, P., Laatikainen, T., . . . Ikaheimo, T. M. (2017). The prevalence of heat-related cardiorespiratory symptoms: the vulnerable groups identified from the National FINRISK 2007 Study. International Journal of Biometeorology, 61(4), 657-668.
55. Pesticide Exposure and Heat Exhaustion in a Migrant Agricultural Worker: A Case of Labor Trafficking, 76, ccm 215-218, Annals of Emergency Medicine (Elsevier B.V. 2020).
56. Pogačar, T., Črepinšek, Z., Kajfež Bogataj, L., & Nybo, L. (2017). Comprehension of climatic and occupational heat stress amongst agricultural advisers and workers in Slovenia. Acta Agriculturae Slovenica, 109(3), 545-554. doi:10.14720/aas.2017.109.3.06
57. Quandt, S. A., Schulz, M. R., Vallejos, Q. M., Feldman, S. R., Preisser, J. S., & Arcury, T. A. (2008). Skin-related quality of life among migrant farmworkers. Journal of Cutaneous Medicine & Surgery, 12(1), 1-7.
58. Raines, N., González, M., Wyatt, C., Kurzrok, M., Pool, C., Lemma, T., . . . Sheffield, P. (2014). Risk factors for reduced glomerular filtration rate in a nicaraguan community affected by mesoamerican nephropathy. MEDICC Review, 16(2), 16-22.
59. Rajewski, P., & Rajewski, P. (2008). Heatstroke - Case report. Family Medicine and Primary Care Review, 10(2), 263-267.
60. Raju, D. S. S. K., Kiranmayi, P., & Vijaya Rachel, K. (2014). Climate change and chronic kidney disease. Asian Journal of Pharmaceutical and Clinical Research, 7(2), 53-57.
61. RiccÒ, M., & Riccò, M. (2018). Air temperature exposure and agricultural occupational injuries in the Autonomous Province of Trento (2000-2013, North-Eastern Italy). International Journal of Occupational Medicine & Environmental Health, 31(3), 317-331. doi:10.13075/ijomeh.1896.01114
62. Riccò, M., Vezzosi, L., Bragazzi, N. L., & Balzarini, F. (2020). Heat-Related Illnesses among Pesticide Applicators in North-Eastern Italy (2017). Journal of Agromedicine, 25(1), 52-64. doi:10.1080/1059924X.2019.1606745
63. Sadiq, L. S., Hashim, Z., & Osman, M. (2019). The Impact of Heat on Health and Productivity among Maize Farmers in a Tropical Climate Area. Journal Of Environmental & Public Health, 2019, 9896410.
64. Sahu, S., Sett, M., & Kjellstrom, T. (2013). Heat exposure, cardiovascular stress and work productivity in rice harvesters in India: implications for a climate change future. Industrial Health, 51(4), 424-431.
65. Sen, J., & Nag, P. K. (2019). Human susceptibility to outdoor hot environment. Science of the Total Environment, 649, 866-875.
66. Smith, D. J., Ferranti, E. P., Hertzberg, V. S., & Mac, V. (2020). Knowledge of Heat-Related Illness First Aid and Self-Reported Hydration and Heat-Related Illness Symptoms in Migrant Farmworkers. Workplace Health & Safety, 2165079920934478.
67. Sorensen, C. J., Butler-Dawson, J., Dally, M., Krisher, L., Griffin, B. R., Johnson, R. J., . . . Newman, L. S. (2019). Risk Factors and Mechanisms Underlying Cross-Shift Decline in Kidney Function in Guatemalan Sugarcane Workers. Journal of Occupational & Environmental Medicine, 61(3), 239-250.
68. Spector, J. T., Krenz, J., & Blank, K. N. (2015). Risk Factors for Heat-Related Illness in Washington Crop Workers. Journal of Agromedicine, 20(3), 349-359.
69. Spector, J. T., Krenz, J., Calkins, M., Ryan, D., Carmona, J., Pan, M., . . . Sampson, P. D. (2018). Associations between heat exposure, vigilance, and balance performance in summer tree fruit harvesters. Applied Ergonomics, 67, 1-8.
70. Stoecklin-Marois, M., Hennessy-Burt, T., Mitchell, D., & Schenker, M. (2013). Heat-related illness knowledge and practices among California hired farm workers in The MICASA Study. Industrial Health, 51(1), 47-55.
71. Trevisan, I. B., Santos, U. D. P., Leite, M. R., Ferreira, A. D., Silva, B. S. D. A., Freire, A. P. C. F., . . . Ramos, D. (2019). Burnt sugarcane harvesting is associated with rhinitis symptoms and inflammatory markers. Brazilian Journal of Otorhinolaryngology, 85(3), 337-343. doi:10.1016/j.bjorl.2018.02.008
72. Vega-Arroyo, A. J., Mitchell, D. C., Castro, J. R., Armitage, T. L., Tancredi, D. J., Bennett, D. H., & Schenker, M. B. (2019). Impacts of weather, work rate, hydration, and clothing in heat-related illness in California farmworkers. American Journal of Industrial Medicine, 62(12), 1038-1046.
73. Wagoner, R. S., Lopez-Galvez, N. I., de Zapien, J. G., Griffin, S. C., Canales, R. A., & Beamer, P. I. (2020). An Occupational Heat Stress and Hydration Assessment of Agricultural Workers in North Mexico. International Journal of Environmental Research & Public Health [Electronic Resource], 17(6), 22.
74. Wegman, D. H., Apelqvist, J., Bottai, M., Ekström, U., García-Trabanino, R., Glaser, J., . . . Bodin, T. (2018). Intervention to diminish dehydration and kidney damage among sugarcane workers. Scandinavian Journal of Work, Environment and Health, Supplement, 44(1), 16-24. doi:10.5271/sjweh.3659
75. Wesseling, C., Aragón, A., González, M., Weiss, I., Glaser, J., Bobadilla, N. A., . . . Barregard, L. (2016). Kidney function in sugarcane cutters in Nicaragua - A longitudinal study of workers at risk of Mesoamerican nephropathy. Environmental Research, 147, 125-132. doi:10.1016/j.envres.2016.02.002
76. Wesseling, C., Aragon, A., Gonzalez, M., Weiss, I., Glaser, J., Rivard, C. J., . . . Johnson, R. J. (2016). Heat stress, hydration and uric acid: a cross-sectional study in workers of three occupations in a hotspot of Mesoamerican nephropathy in Nicaragua. BMJ Open, 6(12), e011034.
77. Wilmsen, C., Castro, A. B., Bush, D., & Harrington, M. J. (2019). System Failure: Work Organization and Injury Outcomes among Latino Forest Workers. Journal of Agromedicine, 24(2), 186-196.
78. Alam Shah, M. A., Inayat, Q., Ali, M., Mughal, I. A., Imdad, L. & Javed, M. (2021). Correlation between occupation and sperm morphology along with sperm count in industrial workers. Pakistan Journal of Medical and Health Sciences, 15(2), 324-326.
79. Alemu Gelaye, K., Debalke, G., Awoke Ayele, T., Fekadu Wolde, H., Sisay, M. M., Teshome, D. F....Daba Wami, S. (2021). Occupational Health Problems among Seasonal and Migrant Farmworkers in Ethiopia: A Cross-Sectional Study. Risk Management & Healthcare Policy, 14, 4447-4456.
80. Butler-Dawson, J., Dally, M., Johnson, R. J., Johnson, E. C., Krisher, L., Sanchez-Lozada, L. G....Newman, L. S. (2020). Association of Copeptin, a Surrogate Marker of Arginine Vasopressin, with Decreased Kidney Function in Sugarcane Workers in Guatemala. Annals of Nutrition & Metabolism, 76(1), 30-36.
81. Diniz, C. O., McKenna, Z., Canuto, L., Magalhaes, F., MacHado-Moreira, C. A., Shibuya, E....Amorim, F. T. (2021). A preliminary evaluation of the kidney function of sugarcane cutters from brazil. Journal of Occupational and Environmental Medicine, 63(2), E53-E58. doi:10.1097/JOM.0000000000002090
82. Grimbuhler, S. & Viel, J.-F. (2021). Heat Stress and Cardiac Strain in French Vineyard Workers. Annals of Work Exposures & Health, 65(4), 390-396. doi:10.1093/annweh/wxaa115
83. Hamerezaee, M., Dehghan, S. F., Golbabaei, F., Fathi, A. & Zamanian, Z. (2020). Comparison of Different Heat Stress Indices for Assessing Farmers' Exposure to Heat Stress. Iranian Journal of Public Health, 49(9), 1810-1812.
84. How, V., Singh, S., Dang, T. & Guo, H. R. (2021). Factors Associated with Health-Risk Perception of Heat Waves among Agroecological and Conventional Farmers in the Tropics. International Journal of Climate Change: Impacts and Responses, 14(1), 45-60. doi:10.18848/1835-7156/CGP/v14i01/45-60
85. Ioannou, L. G., Mantzios, K., Tsoutsoubi, L., Nintou, E., Vliora, M., Gkiata, P....Flouris, A. D. (2021). Occupational heat stress: Multi-country observations and interventions. International Journal of Environmental Research and Public Health, 18(12). doi:10.3390/ijerph18126303
86. Kiatkitroj, K., Arphorn, S., Tangtong, C., Maruo, S. J. & Ishimaru, T. (2021). Risk factors associated with heat-related illness among sugarcane farmers in Thailand. Industrial Health, 24, 24.
87. López-Gálvez, N., Wagoner, R., Canales, R. A., Ernst, K., Burgess, J. L., de Zapien, J....Beamer, P. (2021). Longitudinal assessment of kidney function in migrant farm workers. Environmental Research, 202. doi:10.1016/j.envres.2021.111686
88. Mohammadian, M., Heidari, H., Charkhloo, E. & Dehghani, A. (2020). Heat stress and physiological and perceptual strains of date harvesting workers in palm groves in Jiroft. Work, 66(3), 625-636. doi:10.3233/WOR-203205
89. Pundee, R., Kongtip, P., Nankongnab, N., Anutrakulchai, S., Robson, M. G. & Woskie, S. (2021). Cross-shift change of acute kidney injury biomarkers in sugarcane farmers and cutters. Human & Ecological Risk Assessment, 27(5), 1170-1187.
90. Sandsund, M., Wiggen, O., Holmen, I. M. & Thorvaldsen, T. (2021). Work strain and thermophysiological responses in Norwegian fish farming - a field study. Industrial Health, 05, 05.
91. Silpasuwan, P., Sujirarat, D., Kongtip, P., Inbamrung, S. & Woskie, S. (2019). Hazard Exposure with Health and Safety Outcomes Hinder the Work Ability of Salt Farm Workers in Thailand. Sumerianz Journal Of Medical And Healthcare, 2(10), 125-133.
92. Venugopal, V., Latha, P. K., Shanmugam, R., Krishnamoorthy, M., Omprashanth, R., Lennqvist, R. & Johnson, P. (2021). Epidemiological evidence from south Indian working population—the heat exposures and health linkage. Journal of Exposure Science and Environmental Epidemiology, 31(1), 177-186. doi:10.1038/s41370-020-00261-w
